# Testing the impact of hatha yoga on task switching: a randomized controlled trial

**DOI:** 10.3389/fnhum.2024.1438017

**Published:** 2024-11-05

**Authors:** Bence Szaszkó, Rebecca Rosa Schmid, Ulrich Pomper, Mira Maiworm, Sophia Laiber, Max Josef Lange, Hannah Tschenett, Urs Markus Nater, Ulrich Ansorge

**Affiliations:** ^1^Department of Cognition, Emotion, and Methods in Psychology, University of Vienna, Vienna, Austria; ^2^Department of Clinical and Health Psychology, University of Vienna, Vienna, Austria; ^3^University Research Platform “The Stress of Life—Processes and Mechanisms Underlying Everyday Life Stress”, University of Vienna, Vienna, Austria; ^4^Vienna Cognitive Science Hub, University of Vienna, Vienna, Austria; ^5^Research Platform Mediatised Lifeworlds, University of Vienna, Vienna, Austria

**Keywords:** executive control, mixing costs, task switching, theta, yoga, alpha

## Abstract

Switching attention between or within tasks is part of the implementation and maintenance of executive control processes and plays an indispensable role in our daily lives: It allows us to perform on distinct tasks and with variable objects, enabling us to adapt to and respond in dynamically changing environments. Here, we tested if yoga could benefit switching of attention between distinct objects of one’s focus (e.g., through practicing switching between one’s own body, feelings, and different postures) in particular and executive control in general. We therefore conducted a randomized controlled trial with 98 participants and a waitlisted control group. In the intervention group, healthy yoga novices practiced Hatha yoga 3x a week, for 8 weeks. We conducted two experiments: A purely behavioral task investigating changes in behavioral costs during switching between attentional control sets (74 participants analyzed), and a modality-switching task focusing on electrophysiology (EEG data of 47 participants analyzed). At the electrophysiological level, frequency-tagging indicated no interventional effect on participants’ ability to switch between the auditory and visual modalities. However, increases in task-related frontocentral theta activity, resulting from the intervention, indicated an ability to increasingly deploy executive resources to the prioritized task when needed. At the behavioral level, our intervention resulted in more efficient holding of target representations in working memory, indicated by decreased mixing costs. Again, however, intervention effects on switching costs were missing. We, thus, conclude that Hatha yoga has a positive influence on executive control, potentially through improvements in working memory rather than directly on switching.

**Clinical trial registration:**

clinicaltrials.gov, identifier [NCT05232422].

## Introduction

Executive control refers to the human ability to guide thoughts and action processes through goals and intentions (Miyake and Friedman, 2012). It is, thus, essential for human performance and wellbeing. Executive control encompasses several interrelated higher-order cognitive processes serving goal-directed action (Diamond, 2013; Miyake and Friedman, 2012), adaptation to complex situations (Burgess and Simons, 2005), and the control of behavior (Miller and Cohen, 2001; Miyake et al., 2000). Cognitive flexibility, referring to the ability to adapt human thinking and behavior to changing task demands, rules, or priorities (Diamond, 2013), is one of three core functions summarized under the umbrella term executive control (also called executive functions).

The shifting function, a core facet of cognitive flexibility, is important for regulating and directing attention appropriately—that is, for abandoning responses or processing of features and objects that are currently irrelevant, and for initiating goal-congruent processing or action (Friedman and Miyake, 2004; Miyake et al., 2000). Shifting is implied by focusing attention successively on different features, locations or objects, both within or between tasks. This occurs most powerfully through using different top-down control representations or mental sets in turn (e.g., changes between task-control representations for visual search of different target colors or for monitoring of distinct actions). The shifting function, thus, enables humans to perform effectively on multiple tasks and to adapt to changing rules or information in a timely manner (Kiesel et al., 2010).

### The effect of yoga on shifting

Importantly, there is growing evidence that certain physical and mental exercises benefit executive functions (e.g., Diamond and Lee, 2011; Esmaeili et al., 2023; Guiney and Machado, 2013; Xue et al., 2019). Among them is yoga, a holistic approach, encompassing a number of practices of Indian origin that is clearly and explicitly directed at the physical and mental realm simultaneously (Feuerstein, 2012). Here, we looked at Hatha yoga because of its popularity, increasing the potential impact of our findings. Hatha yoga emphasizes combining physical and mental exercises, potentially setting it apart from other, more puristic mental (e.g., meditation) or physical (e.g., running) activities. Hatha yoga includes different forms of stretching, changing postures, as well as mindful breathing and imagination (Shapiro et al., 1998). Past research investigating yoga’s effect on executive functioning has mainly focused on inhibition (e.g., Luu and Hall, 2017; Oken et al., 2004, 2006; Sandroff et al., 2015; Szaszkó et al., 2023; Telles et al., 2013), working memory (Bowden et al., 2012; Danilewitz et al., 2021; Kyizom et al., 2010; Telles et al., 2012), or both (Gothe et al., 2013; for a review on Yoga’s effects on executive functions, see Luu and Hall, 2016). In the current study, we, thus, looked at the impact of a yoga intervention on the shifting function, aiming to close a knowledge gap in yoga’s effects on the distinct executive functions. Previous evidence suggests that yoga may influence the shifting function both acutely (Gothe et al., 2013, 2016; Luu and Hall, 2017) and for extended times (Telles et al., 2013; Vhavle et al., 2019; but cf. Fritz and O’Connor, 2022; Oken et al., 2006). However, very few randomized controlled trials have been conducted regarding this question, with most research using clinical or otherwise potentially compromised samples (like sedentary older adults). Our study is one of the first to examine such potential changes in a healthy sample, thereby providing an opportunity to test the hypothesis under more conservative conditions, with less room for improvement from a better pre-intervention level of executive functioning.

Possible reasons for the beneficial effects of yoga on the shifting function include improvements in executive functioning in general through the attentive nature of yoga, where one is encouraged to focus on one’s own body, thoughts, feelings, and, in general, the present moment (Schmalzl et al., 2015), as well as a direct training of the shifting function in particular through the shifts of attention during yoga (e.g., from one posture to another or from one object to another) promoting cognitive flexibility relatively directly (Bransford et al., 1999). In addition, the potential stress- and anxiety relieving effect of yoga, achieved through parasympathetic activation (Zaccaro et al., 2018), may also liberate resources for executive control that would otherwise be bound (e.g., by rumination; cf. Derakshan and Eysenck, 2009; Liston et al., 2009; McRae and Gross, 2020).

To examine whether yoga has an effect on the shifting function, in two experiments, we looked at distinct scenarios and compared the differences in participants’ performance, pre- to posttest, on experimental paradigms evaluating these different types of task-switching. To note, our study is the first to compare the impact of the intervention on different types of shifting: within-modality shifting between representations of searched-for target colors (here: red and green) in a visual search task (Experiment 1), and between-modality shifting of auditory and visual modalities (Experiment 2). In this way, we can test for convergent evidence of shifting improvements across Experiments 1 and 2 and, thus, control for the more specific side conditions of each specific test.

### Experiment 1: Switching between task sets of target colors

In Experiment 1, based on the experimental design of Büsel et al. (2019), we wanted to know if yoga improves participants’ ability to switch between two target colors. Here, we looked at two types of behavioral costs: those of participants’ switches between two distinct task sets for different target colors in subsequent trials (*switching costs*), which typically manifest, among other things, in longer response times in trials in which participants are required to switch to a new task set (Monsell, 2003), and costs of sequentially performing multiple tasks versus performing a single task (*mixing costs*), which typically manifest, among other things, in longer response times in trials in which multiple tasks have to be attended simultaneously (e.g., Rubin and Meiran, 2005).

If yoga improved switching performance, in Experiment 1, we expected that the intervention decreased switching costs and mixing costs compared to the control group (H1). In the present study, performing multiple tasks did not mean searching for two different target colors simultaneously, but rather having to search for one of two target colors that could change or repeat from one trial to the next (as opposed to searching for a single target color across all trials).

### Experiment 2: Switching between distinct sensory modalities

In contrast to Experiment 1, in Experiment 2, we investigated whether the intervention influenced neural responses associated with shifting between auditory and visual modalities (while ignoring the currently unattended modality). To this end, we developed a task that not only facilitated task switching but also resulted in a performance benefit when switching. Typically, switching between tasks slows responses and decreases accuracy. These switching costs imply that, for the participants, there is not much incentive to willingly switch a task. This is even the case with cued switches (so-called “residual switch costs,” e.g., Monsell, 2003). In the current Experiment 2, however, we ensured participants’ willingness to switch tasks by providing a relatively substantial performance benefit for the switches: Participants are likely to willingly switch between tasks and happily drop the ongoing task because a task switch means that the modality-specific information currently held in working memory can be dropped with immediate effect. To note, this should mitigate switching costs, thereby making them unreliable as a change measure of the switching function. Additionally, we also wanted to use a more ecologically valid task with concurrent information in both auditory and visual modalities compared to tasks including traditional switch vs. repeat trials; however, this would have made behavioral costs even less interpretable. Therefore, unlike in Experiment 1, we do not report switching (and mixing) costs in Experiment 2, focusing solely on electrophysiological outcomes. This task used a bimodal (auditory and visual) cue to signal transitions between auditory and visual tasks, with the major trade-off of not being able to fully consider performance-related costs, such as switching and mixing costs, unlike in the present Experiment 1. However, this approach had two clear benefits: Firstly, it allowed us to separate the effects related to memory from those associated with task switching. Typically, recall of a second task control representation during task shifting increases memory load at times of a shift. However, in our protocol, switching away from one task eased the load on participants’ working memory for the currently abandoned task, freeing up resources at the times of shifting. Thus, we avoided the typical confound of higher versus lower memory load and switching versus repeating of a task. Secondly, the use of this specific cue actively encouraged participants to switch between modalities, thereby increasing internal validity—that is, increasing the likelihood that the participants used the cue for task shifting—, while additionally minimizing the probability of false alarms due to attending to the wrong modality.

For examining switching-related effects of the intervention, we used frequency tagging through steady-state potentials (Norcia et al., 2015; Picton et al., 2003; Vialatte et al., 2010), where we used distinct frequencies for both auditory (24 Hz) and visual (20 Hz) presentation modalities so that the currently prioritized modality input resulted in higher spectral power at the respective stimulation frequency, and in the case of the SSVEP, its second harmonic (40 Hz), than the nonattended one. This enabled us to study neural activity associated with the distribution of attention between distinct modalities, as well as their respective changes through the intervention (Saupe et al., 2009; Talsma et al., 2006). Following the intervention phase, we expected higher (vs. lower) spectral power differences in the tagging frequency—or, in case of the SSVEP, its second harmonic (40 Hz)—of the attended (vs. unattended) modality in the intervention group versus the control group, relative to the pretest baseline differences (H2a). Looking at the period directly after a modality switch, we also expected changes in power at the respective tagging frequency or their second harmonic (H2b).

Additionally, we looked at a single oscillatory frequency band known to be critical for optimal task switching: Theta (4–7 Hz) oscillations within medial frontal cortex. Increased frontoparietal connectivity and spectral power in the theta range, particularly in midfrontal regions, has been repeatedly linked to enhanced cognitive flexibility and executive functioning (Cavanagh et al., 2012; Gevins et al., 1997). These oscillations also reflect diverse memory-related processes vital for task switching: the reactivation of task set memories (Gladwin and de Jong, 2005), successful manipulation of working memory content (Itthipuripat et al., 2013; Karayanidis et al., 2009), and facilitated encoding, retrieval, and transfer between memory systems (Klimesch et al., 1994, 2001; Rizzuto et al., 2006; Sauseng et al., 2002). Such (working) memory processes are integral to task switching because maintaining different relevant task-control representations in memory is essential to swiftly switch between them if the conditions require this. In line with this reasoning, increased theta activity and connectivity are associated with task switching (Cooper et al., 2015; Sauseng et al., 2006) and theta activity, originating in medial cingulate cortex is a key to maintaining task-control representations (Cohen et al., 2008; Cooper et al., 2017). Neurofeedback studies even suggest a causal role of midfrontal theta in switching performance (Enriquez-Geppert et al., 2014). Other theories posit that an increase in midfrontal theta could also reflect increases in inhibitory control, potentially encompassing both response and sensory inhibition processes: In Go/NoGo-paradigms, refraining from responding has repeatedly led to an increase in frontocentral theta (e.g., Nigbur et al., 2011; Beltrán et al., 2019); for sensory inhibition first data from mice (Siegle and Wilson, 2014) and humans (Snipes et al., 2022) support the notion that theta is involved in (more active) sensory inhibition or (more passive) cortical disengagement that serve the purpose of compensation for cognitive load in states of high conflict. Regardless of whether the increase in midfrontal theta reflects a more general enhancement in executive control, specific changes in shifting, or heightened inhibition of irrelevant sensory information and response inhibition following a shift to another modality, the result would be an overall increase in theta activity. Thus, we hypothesized that if the yoga intervention increased task switching or altered executive control processes in general (i.e., even in case that H2 could not be confirmed), we expected increased clustered midfrontal theta activity in response to a switch (H3) in the intervention (relative to the control) group in the posttests relative to pretest baseline. Exploratory spectral analyses of theta aiding the process of finding an answer to H3 are also reported in the results.

Finally, we intended to take an explorative look at the spectral power in the alpha band (8–12 Hz); multiple studies have reported increases in alpha-band activity through yoga (Ajjimaporn et al., 2018; Bharadwaj and Kulshrestha, 2013; Trakroo et al., 2013; for a review, see Desai et al., 2015). Increased alpha band (8–12 Hz) activity has been shown to be related to increased relaxation ability (Stancák Jr and Kuna, 1994; Trakroo et al., 2013), as well as to improved gating of sensory input, implying reduced processing in sensory areas (Zumer et al., 2014). This can be particularly beneficial when information irrelevant, or even detrimental, for optimal task performance, or negatively impacting mental health, can be ignored. Thus, improved inhibition can, in the present case, aid executive control as well as switching performance.

## Methods

### Participants

Three hundred and forty-five participants were assessed for eligibility between December 2^nd^, 2021, and April 27^th^, 2022. For the exact allocation of places on the waiting list, see Szaszkó et al. (2023). One hundred and sixteen participants were admitted to the trial, out of which 98 participants (*N*_Intervention_ = 54; 83 female; *M*_Age_ = 25.03 years; *SD*_Age_ = 4.18 years; range 18–40 years) were part of the study at the start of the intervention. For baseline characteristics, see Table 1.

**Table 1:** Baseline characteristics of the intention-to-treat population (from Szaszkó et al., 2023).

	Total (*n* = 100)	Intervention (*n* = 44)	Control (*n* = 56)	*p*
Age (in years)				0.316
	25.12 ± 0.42	24.73 ± 0.72	25.61 ± 0.50	
Gender (*n* = 99)				0.673
Women	81 (81.8%)	45 (81.8%)	36 (81.8%)	
Men	18 (18.2%)	10 (18.2%)	8 (18.2%)	
Working hours per week				0.872
0	22 (22.0%)	12 (21.4%)	10 (22.7%)	
1–20	52 (52.0%)	30 (53.6%)	22 (50%)	
20–40	23 (23.0%)	13 (23.2%)	10 (22.7%)	
Over 40	3 (3.0%)	1 (1.8%)	2 (4.6%)	
Smoking (at least occasionally)				>0.999
Yes	29 (29.0%)	16 (28.6%)	13 (29.6%)	
No	71 (71.0%)	40 (71.4%)	31 (70.4%)	
Alcohol use (at least occasionally)				>0.999
Yes	84 (84.0%)	47 (83.9%)	37 (84.1%)	
No	16 (16.0%)	9 (16.1%)	7 (15.9%)	
Physically active				>0.999
Yes	70 (70.0%)	31 (70.4%)	39 (69.6%)	
No	30 (30.0%)	13 (29.6%)	17 (30.4%)	
Mindfulness practice (but not yoga)				0.991
Yes	17 (17.0%)	9 (16.1%)	8 (18.2%)	
No	83 (83.0%)	47 (83.9%)	36 (81.8%)	
Depression (PHQ-9; 0–27)	7.09 ± 0.44	7.53 ± 0.61	6.52 ± 0.62	0.248
BMI	21.91 ± 0.29	21.71 ± 0.36	22.18 ± 0.48	0.430
Somatic symptoms (PHQ-15; 0–30)	6.53 ± 0.45	6.73 ± 0.67	6.27 ± 0.60	0.611
Stress (PHQ; 0–20)	4.43 ± 0.30	4.41 ± 0.47	4.45 ± 0.40	0.943

Data are presented as mean ± SD or *n* (%). BMI: Body-Mass-Index. PHQ: Patient Health Questionnaire. Three subscales of the PHQ are reported here: PHQ-9 assessing depression, PHQ-15 assessing somatic symptoms, and 10 specific questions of the PHQ assessing stress. p values are calculated from independent samples *t*-tests for continuous variables, and Chi-squared tests for categorical variables.

**Figure 1:**
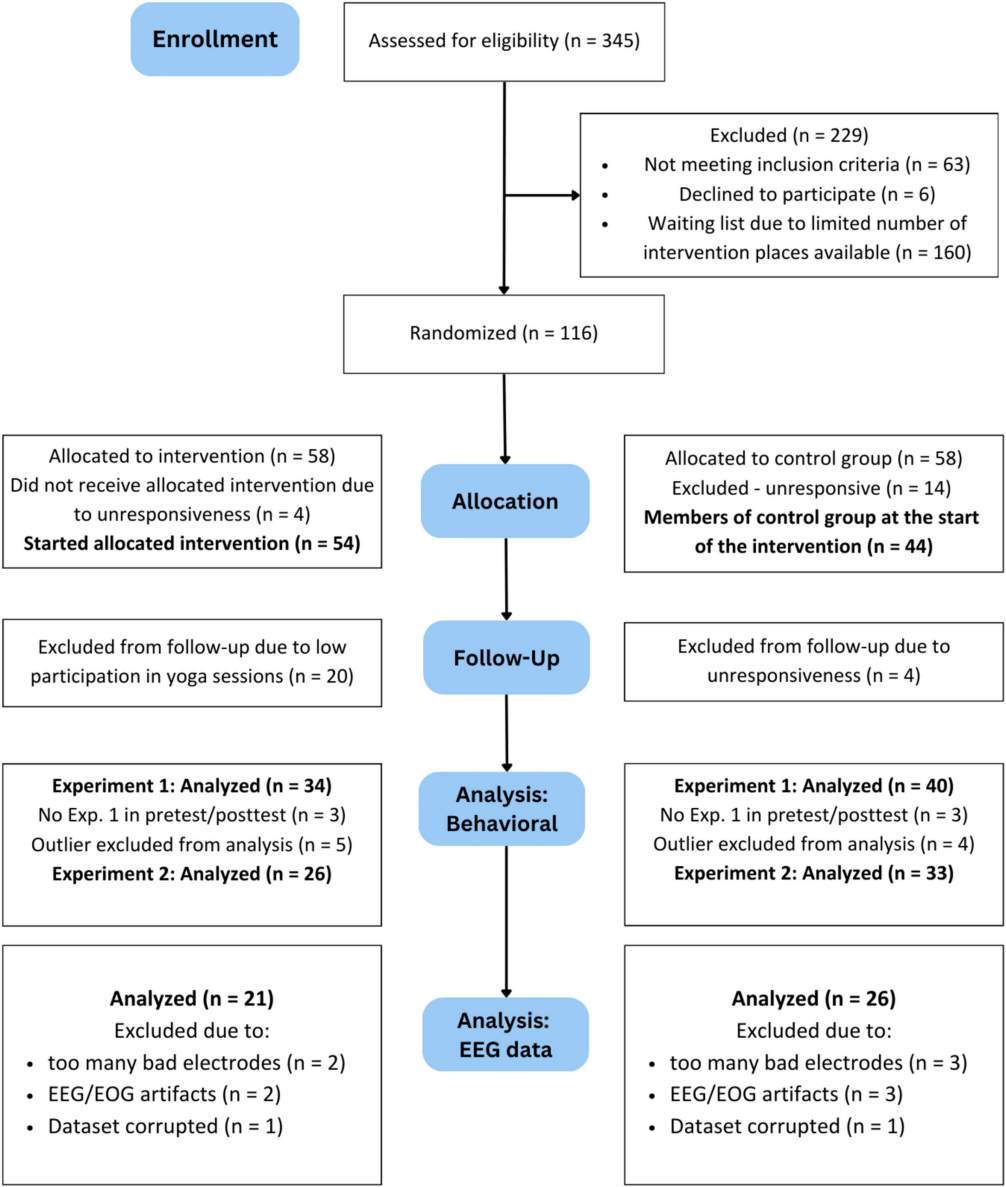
Consort chart of the study. Central column: Trial profile, including analyses. Left and right columns: Reasons for exclusion after the previous analysis step are listed below the number of analyzed participants for each step. When participants were excluded from behavioral analyses, their EEG data was also not analysed. EEG, electroencephalography; EOG, electrooculogram.

Our intended sample size was 102 in order to achieve 80% power with *d* = 0.5 and an alpha error probability of 0.05, not only for our EEG data (which typically result in very high power) and experiments, but also the questionnaires used. Figure 1 shows the trial profile of the study in form of a CONSORT chart. We recruited participants via the University of Vienna’s Laboratory Administration for Behavioral Sciences (LABS) platform and the Vienna Cognitive Science Hub’s Study Participant Platform (SPP), and by advertisement through flyers and social media.

Next, we screened potential participants with a shortened version of the Diagnostic Interview for Mental Disorders (Mini-DIPS; Margraf, 1994). If subjects showed possible current or past psychiatric or psychological disorders, they were excluded from the study. We did this for several reasons: First, focusing on a non-clinical, general, and healthy sample helps to enhance the generalizability and external validity of our findings. Studies on the impact of yoga on specific populations are abundant (Bilderbeck et al., 2013 for prison inmates; Gothe et al., 2014, 2016 for older adults; Oken et al., 2004, for patients with multiple sclerosis), and we aimed to contribute insights relevant to the general population, even if this meant we measured the impact more conservatively (i.e., in a group benefitting less because they were already good at task shifting and executive control). Second, assessing cognitive abilities in individuals with psychiatric or psychological disorders can be challenging due to the potential confounding effects of their conditions. Individuals with such disorders may also have different baseline cognitive abilities, making it difficult to attribute any observed changes solely to the intervention.

Participants that had practiced yoga more than twice a month for the last 12 months, those that consumed psychopharmaceuticals regularly, as well as those with alcohol or drug abuse were also excluded. In addition, only participants between 18 and 40 years of age were admitted. Participants were also excluded if they had below level C1 German proficiency, uncorrected impaired vision or hearing, or had suffered a skull fracture or concussion within the past 6 weeks. For legal reasons at the time of the study, lack of proof of COVID-19 recovery or vaccination also led to exclusion from the study.

The starting date of our intervention was pre-set, and pretesting laboratory capacity was limited. Therefore, whenever a participant opted out during the pretesting phase, substitutes from the waiting list were invited to participate in the experimental group instead. However, sometimes no fitting testing slots were available anymore. Thus, already from the outset, the total number of participants was lower than intended.

To compare against the intervention, we used a control group not taking part in the intervention. This comparison is maximally sensitive to any beneficial effect the yoga intervention might have because the waiting list group did not receive any treatment at all before posttests were administered. However, using this approach also meant that double-blinding was not possible. It was clear to participants if they were participating in a yoga intervention or not (the waitlist control group). The same was true of the yoga teachers. They were aware that they were teaching yoga classes. However, participants and yoga teachers were naïve with respect to the hypotheses of the current study, and yoga teachers were not involved in the analyses of the data. In addition, a pre-post comparison of participants’ performance in different cognitive tasks meant that participants were instructed to perform as well as they could at each measurement time point. Thus, forging of the hypothesized intervention-dependent performance difference between pre- and posttest was unlikely and would have required sophisticated knowledge and strategies on the side of the participants (e.g., participants’ holding back during pretest measurement and giving it their best at posttest measurement in the intervention group based on their expectations of what we would have hypothesized).

During the first pretest session, participants were briefed on the procedure and risks of the study and gave their written informed consent. After this session, participants were assigned to a group using block randomization (block size: four; custom Python script written and executed by the first author). All participants were debriefed in the second posttest session. Participation in pretests / posttests was compensated with a payment of 30 EUR each, with a bonus payment of 10 EUR (20 EUR) for participation in an average of four (five) classes per week. There were no adverse experiences associated with the intervention or the waitlist reported by any of the participants throughout the duration of the intervention; one participant had to abort Experiment 1 because she did not feel well during practice trials.

### Study design and intervention

The present experiments were part of a larger study with a parallel design investigating the effect of a Hatha yoga intervention on stress, anxiety, and the executive function of suppression. Data collection began in January 2022 and ended in August 2022. In the course of this study, we used questionnaires, behavioral, electrophysiological, and biological markers. Detailed results on these measures, as well as experimental and electrophysiological results on the inhibition function can be found in Szaszkó et al. (2023). The present study focuses mainly on Hatha yoga’s effect on the shifting function; the results concerning hormonal stress markers and their relations to stress, anxiety, and cognitive functioning can be found in Szaszkó et al. (2024).

After preselection, participants were pseudo-randomly assigned to an intervention- and a waitlisted control group, to ensure equal group sizes. The intervention group was split into two cohorts. In the first pretest session, participants gave their informed consent, were scanned for colorblindness using an Ishihara test, and completed a task in which they had to switch between different attentional control sets (experiment 1). In the second pretest session, participants were trained in the collection of saliva samples, completed a suppression task (Szaszkó et al., 2023), and the second switching task, assessing participants ability to switch between sensory modalities (experiment 2). All tasks throughout both sessions were completed in the laboratory of the University of Vienna. The days after pretests, participants submitted their saliva samples and completed online questionnaires. Participants in the intervention group were required to attend at least three 60-min yoga classes per week, for a total of 8 weeks. Subjects were excluded if they failed to participate at least twice for 3 consecutive weeks. This led to an average participation of 2.55 sessions per week (*SD* = 0.55).

Classes were taught privately by four professional yoga instructors at the “FREIRAUM” studio in Vienna. Table 2 shows the focus of each week’s classes. Online pre-registration was required to track attendance, which was then double-checked by the yoga teachers at the beginning of class. Following the 8-week intervention, participants took part in two posttest sessions: In the first posttest session participants again completed the task for experiment 1, in the second posttest session they again completed the suppression task, the task for experiment 2, and received a debriefing. Between posttest sessions, they completed the same questionnaires they had previously completed and again collected saliva samples. Participants in the control group underwent the same process as those in the intervention group, except for the yoga intervention. Once posttests were completed, they were, however, given the opportunity to participate in yoga classes free of charge for a duration of 8 weeks.

**Table 2:** Focus of yoga sessions per week and exemplary asanas (yoga postures; from Szaszkó et al., 2023).

Week	Focus	Exemplary asanas
1	Sun salutation, breathing exercises	Downward Facing Dog, Cobra Pose, Sprinter Pose
2	Standing positions	Warrior I and II, Triangle Pose, Chair Pose
3	Forward Bends	Different variations of Forward Bends, Pigeon Pose, Butterfly Pose
4	Twists	Waist Turn, Crocodile Pose, twisted Triangle Pose
5	Backbends	Shoulder Bridge, Locust Pose, Bow Pose
6	Sitting poses	Lotus positions, Boat Pose, Child Pose
7	Balance	Tree Pose, Warrior III, Crow Pose, Half Moon Pose
8	Combining focuses 1–7	Combinations of the previous exemplary asanas

In each week of the Hatha Yoga intervention, comprising a total of 8 weeks, a different focus was set, which determined the practiced asanas (yoga postures) of that week. This focus had to be applied by all yoga teachers when teaching their respective individual yoga sessions.

We used a per-protocol (PP) approach to analyze the data. The per-protocol approach includes only those participants who adhered strictly to the intervention protocol, excluding those who did not complete the intervention as specified. This contrasts with the intention-to-treat (ITT) analysis, which includes all participants as originally allocated, regardless of whether they completed the intervention according to the protocol.

The per-protocol approach was chosen for several reasons. First, it provides a clearer picture of the intervention’s efficacy by focusing on participants who fully engaged with the yoga practice as intended. This is particularly important for understanding the potential benefits of yoga in a controlled setting. Second, by excluding non-compliant participants, we can better assess the true effect of the intervention without the noise introduced by partial adherence, which is crucial when studying a healthy population where the expected effect sizes might be smaller. Albeit the PP approach is more vulnerable to self-selection bias (Andrade, 2022) and to the magnification of pre-existing motivational differences (Gupta, 2011) than ITT analysis, it does not require data imputation. By only considering the actually observed data of participants that complied with the instructions of the experimental tasks and the requirements of the intervention protocol, the PP approach navigates around the pitfalls of large numbers of imputed data, thus, reflecting more accurately the interventions’ impact.

Although this approach might also reduce the generalizability of the findings compared to an ITT analysis, as well as reducing the power of the study, it enhances internal validity and allows us to draw more precise conclusions about the intervention’s effectiveness. By imposing strict exclusion criteria and including only those participants who rigorously followed the intervention schedule, we ensured that the data accurately reflects the intervention’s impact (Green et al., 2019). This methodological choice aligns with our objective to assess the efficacy of yoga practice in a well-defined healthy sample, providing a robust test of our hypotheses within this context (Tripepi et al., 2020).

### Experiment 1: Switching attention between colors

#### Task design

In Experiment 1, participants had to search for a color-defined target ring and report the orientation of a letter *T* within the target. We intended to replicate the results by Büsel et al. (2019), showing that participants indeed set up distinct task sets (here, attentional control settings) for different target colors and switch between them, rather than the same unchanging task set during the entire experiment, thereby validating the experimental design before investigating the effects of the yoga intervention on behavioral costs. For this, two conditions must be fulfilled: First, the presence of cueing effects with top-down matching cues, indicating that this cue attracted attention at all, where performance in valid trials (cue and target at same position) is significantly better than performance in invalid trials (cue and target at different positions) only in case of the color of the cue matching the color of the target. The results of these tests are reported in Appendix A. Second, more importantly, we tested for the presence of behavioral costs as addressed briefly in the Introduction and reported in the Results section. To measure switching costs (for both RTs and ARs), we used a two-color search task and compared performance between trial-by-trial target-color switches (e.g., between a green target in Trial *x* and a red target in Trial *x* + 1) versus trial-by-trial target-color repetitions. (2) To measure mixing costs, we compared performance (again for both RTs and ARs) between trials where participants searched for two potential target colors as compared to trials where participants searched for one target color, with trial-by-trial target color repetitions only. Note that we partly deviated from the original definitions of switching and mixing costs because, in the present case, we tested for shifting between distinct attentional control sets (here: the two colors) within the same nominal task rather than clearly separate tasks (as initially implied by switching costs), as well as performance decrements caused by two potential target colors (and thus holding two sets in working memory) compared to one, rather than comparing the execution of two tasks to one (as initially implied by mixing costs). Our comparisons are based on the assumption that switches between control representations within a nominal task are theoretically related to dual-task execution and that these related phenomena should not be artificially separated by misleadingly narrow definitions of what defines a task (Kleinsorge and Heuer, 1999; Ort et al., 2017; Yeung and Monsell, 2003).

While EEG was measured in Experiment 2, this was not done in Experiment 1. We opted for the sole collection of behavioral data in Experiment 1 for two main reasons: First, the protocol (a replication of Büsel et al., 2019) had already been proven to assess behavioural costs precisely without the need for additional methods, thus allowing conclusions to be drawn about changes in the switching function. Second, in the protocol of Experiment 1, the typical preconditions for frequency analyses of EEG were not met. In particular, cue-target intervals are relatively short in this protocol, but the Fourier transform requires a substantial cue duration. Therefore, we opted against EEG recording for Experiment 1.

#### Procedure

The experiment consisted of four blocks containing 240 trials and 24 practice trials each, and two distinct blocked conditions: a single-color condition and a dual-color condition. Blocked conditions (single- vs. dual-color) were either A-B-A-B or B-A-B-A, balanced across participants. In the single-color condition, the target was always of the same color defined at the beginning of the block (red or green). In the dual-color condition, in each trial, the target could unpredictably take on one of two colors (red or green, within blocks), with both instructed as target-defining colors at the start of the block.

**Figure 2:**
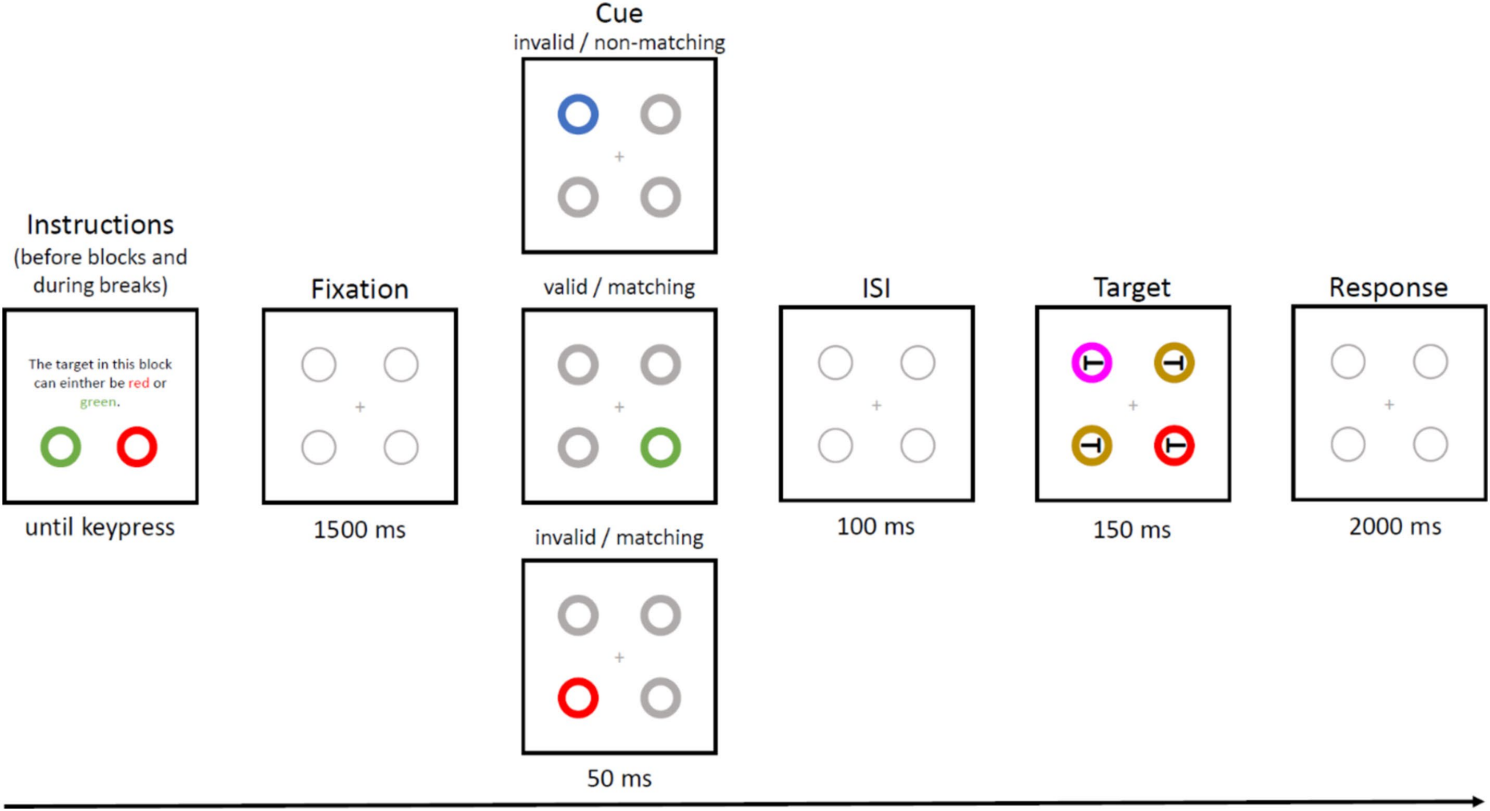
Exemplary trial of Experiment 1. Taken from Büsel et al. (2019). Blocks began with an instruction display showing possible target colors, which, in single-color blocks, were red or green (in different blocks, one block with red, one with green color), and in dual-color trials (depicted here) were red and green, but only one of these colors was randomly assigned as the target color in each trial. After a fixation display, a cueing display followed, with as singleton-color cue in a unique color either matching (same color as one of the potential target colors) or not matching (color different from all potential target colors) the task set. Cues could be presented at target position (valid trial) or away from the target (invalid trial). After a fixed interstimulus interval, the target display with four black *T*s was presented. *T*s were tilted clockwise or counter-clockwise with equal probability, and surrounded by colored rings, one of which was the target color. Participants indicated the direction of the tilt (here, counter-clockwise) inside the target ring by keypress.

Figure 2 illustrates an exemplary trial. The experiment started with an instruction screen, in which participants were instructed about the target color(s) of the block. In each trial, participants first saw a grey fixation cross presented for 1 s, followed by a cueing display with four rings for 50 ms. One of the rings in the display was a color singleton (the cue).

After an interval of 100 ms following the cueing display, the target display was presented for 150 ms. The target display consisted of four rings. All of them were colored, one of them in the target color. All circles in the target display contained a horizontally tilted *T*. Participants reported the orientation of the target *T* by pressing Key “y” to a counter-clockwise and key “m” to a clockwise tilted *T*. Participants were instructed to respond as fast and accurate as possible. In two-color blocks, the target color changed with 0.5 probability from trial *n* to *n* + 1, yielding equal numbers of trial-by-trial target-color changes and repetitions. Feedback was given on screen for 500 ms after wrong responses (the message “wrong” was displayed) and after response times exceeding 1,200 ms (“respond faster” was displayed).

#### Apparatus and stimuli

The experimental task was presented on a 24.5” G2590PX AOC Gaming LCD monitor (with 54.4 cm x 30.3 cm visible), with a refresh rate of 100 Hz. Responses were logged using a QWERTZ-keyboard. Viewing distance was held constant at 57 cm using a chin-forehead-rest. OpenSesame (Version 3.3.10; Mathôt et al., 2012) was used to present the task.

All colors in the current manuscript are provided in CIELAB color space (Schwiegerling, 2004) and are equally bright (L* = 70). The fixation cross was 0.6° × 0.6° large, placed at the centre of the screen, and. Presented against a black background (L* = 8, a* = 23, b* = −27). The cueing display contained four gray (L* = 70, a* = −4, b* = 1) rings (outline width: 0.1°; radius: 1.2°) with a 2.7° eccentricity, with the rings presented on the corners of an imagined square centered on the screen. Cues took on either the target color (red: a* = 65, b* = 56; or green: a* = −47, b* = 48; in single-color blocks) or one of the target colors (in dual-color blocks), which all constituted instances of top-down matching cues (as they matched a task set); alternatively, the cue took on another color, constituting a nonmatching—blue (a* = 25, b* = −85)-cue. Matching and nonmatching cues were equally likely, and participants did not know in advance which cue was presented. For each trial, one cue appeared at the location of the target (valid trials) or at a location away from the target (invalid trial). Across trials, cue and target positions were uncorrelated. Therefore, the cues were not predictive of the target locations. In the target display, besides the (red or green) target ring, two rings were yellow (a* = −5, b* = 49), and one ring was either magenta (a* = 75, b* = −69) or cyan (a* = −29, b* = −13), with equal probability. The orientations of the *T*s shown within the rings were pseudo-randomly distributed across all four possible stimulus locations, with two of them tilted to the left and two of them tilted to the right in every trial.

#### Data analysis

Behavioral data (of both experiments) was analyzed using RStudio (Version 1.4.1717; RStudio Team, 2021) with R (version 4.1.2; R Core Team, 2021), based on the packages data.table (Version 1.14.2; Dowle and Srinivasan, 2021), ez (Version 4.4.-0.; Lawrence, 2016), rstatix (Version 0.7.2, Kassambara, 2023), schoRsch (Version 1.9; Pfister and Janczyk, 2020), and tidyverse (Wickham et al., 2019). We excluded participants using a generalized Extreme Studentized Deviate (ESD) sensitive for multiple outliers (Rosner, 1983) with an alpha level of 0.05. In Experiment 1, we analyzed participants’ response times (RTs) and accuracy rates (ARs). For RT analysis, individual trials in which participants’ RTs exceeded ±2.5 *SD*s from their individual mean RTs were removed. This was done to ensure that mean performance was representative for each condition and not distorted by extreme outliers. Where indicated, we corrected for multiple comparisons using the Holm-Bonferroni method (Holm, 1979). Trials in which cues and targets appeared on the same location were excluded from the analysis to prevent location-priming effects.

The effects of the intervention were calculated by subtracting posttest from pretest values and by comparison between groups using independent-samples *t*-tests (one-sided only in case of directional hypotheses).

### Experiment 2: Task switching

#### Apparatus, stimuli, and procedure

Participants completed the experimental task using a 19” CRT monitor (Sony Multiscan). The monitor had a resolution of 1,280 × 1,024 pixels and an aspect ratio of 4:3. The refresh rate was 100 Hz. PsychoPy 3 (Peirce and MacAskill, 2018) was used to present the task. The distance between participants’ eyes and the screen was 57 cm and kept constant by a chinrest. A conventional QWERTZ-keyboard was used for collection of participants’ responses. The experiment took place in a soundproof, dimly lit room. Participants had to complete a two-back task, in which they were concurrently presented with stimuli at a consistent rate in the visual (20 Hz) as well as in the auditory (24 Hz) domain, using modality-unique tagging frequencies. The experiment itself did not consist of distinct trials, but of 15 blocks of 180 s of auditory and visual stimuli presented continuously and concomitantly, with self-paced breaks in between blocks. By doing so, we refrained from the traditional combination of switch- versus repeat trials (including repetition cues), using our experiment in a more ecologically valid framework with concurrent information presented for a varying amount of time, with high temporal ambiguity regarding possible switches. This meant that direct comparisons of switching and repetition performance are difficult to interpret. Remember, however, that our focus was on the evaluation of the (electrophysiological) outcomes of switches to either one or to the other modality, as well as the related interventional effects.

**Figure 3:**
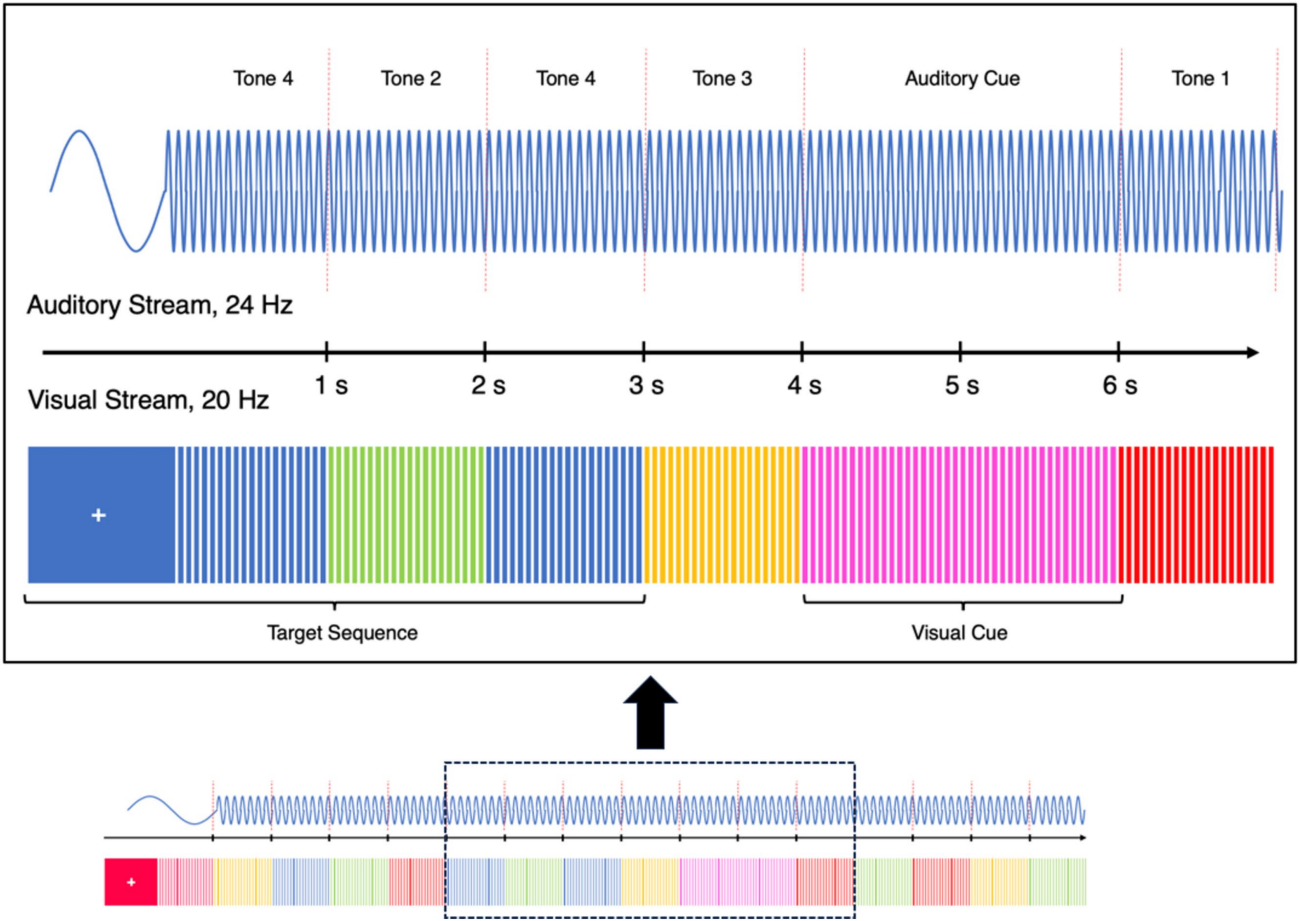
Schematic illustration of part of an exemplary block in Experiment 2. Depicted is the bimodal n-back task in Experiment 2, during which participants had to detect a certain pattern (a repetition of the color blue/the highest tone with one stimulus in-between) in the respective to-be-attended-to modality. Upper row: The blue sine wave in the auditory domain represents sequential stimulation with six distinct tones of equal durations (1 s each) but of distinct frequencies (Tones 1 to 4 + one other frequency that was used for the cue) that each were presented 24 times per second. Red dotted vertical lines indicate a change between subsequent tones occurring every second (with the exception of the cue that lasted for 2 s). The auditory cue consisted of a single pure tone that lasted for 2 s and indicated a switch of the to-be-attended-to modality. Middle row: Here, the rectangle with the fixation cross and the colored lines to the right of it represent stimulation by six consecutively presented and differently colored rectangles in the visual domain at the center of the screen, each presented 20 times per second, with colors switching every second (again with the exception of the cue). Colors switched every second and in synchrony with the auditory stimuli. An exception to this rule is the visual cue (one of two colors, depending on the to-be-attended-to modality), which lasted for 2 s. The first set of blue stimuli, followed by a second set at temporal position n + 2 represents the target sequence in the visual modality to which participants were asked to respond. The set of pink stimuli represents the visual cue which, together with the synchronously presented auditory cue signaled that the other modality was now to be attended to. Lower row: A depiction of how a block of a specific modality was embedded in preceding and subsequent blocks of the other modality.

After having been cued by a switch cue (present in both, the visual and auditory modality), indicating that participants should switch from vision to audition or vice versa, participants had to compare the current input from one modality to that of a stimulus two positions back in time in the same modality. Figure 3 illustrates a part of an exemplary block.

Stimuli in the visual domain consisted of four differently colored equiluminant (CIE L*a*b*, L* = 60) rectangles (orange: a* = 61, b* = 71, blue: a* = 12, b* = −71, red: a* = 80, b* = 69, green: a* = −54, b* = 55) at screen center on a black (L* = 3.3, a* = 0.1, b* = −6.5) background and changed color once per second, while flickering at 20 Hz. We used a black background to increase contrast and, thereby, salience of the visual stimuli. The visual target was a pattern of a blue rectangle followed by another blue rectangle, with one differently colored stimulus in-between (i.e., a two-back task). In the auditory domain, a total of six pure tones (at frequencies of 311, 440, 622, 880, 1,244, and 1,760 Hz, with a sinusoidal waveform) served as stimuli. Analogous to the visual task, the auditory target was a pattern of a 1,760 Hz tone followed by another 1,760 Hz, with one different tone in-between. Participants were informed about the identity of the target patterns before the experiment had started. Auditory and visual targets could not appear at the same time. The number of targets followed a Gaussian distribution, ranging from three to eight targets per block and modality. If a stimulus had the characteristic N in Trial *X*, it could not assume the same characteristic N in the following Trial *X* + 1 (i.e., if a visual stimulus was blue in Trial *X*, it could not be blue again in Trial *X* + 1). Sound volume was adjusted to individually comfortable levels. Subjects were required to respond to the described N-2 target patterns in both the visual and auditory condition by pressing the “down arrow” key as fast and accurate as possible.

At the beginning of the experiment, participants completed a 3-min practice block, which they could repeat if necessary. Before and after the experimental blocks, participants also completed two 1-min baseline blocks where only stimuli in one modality were presented. These four blocks (two auditory, two visual) were used to calculate a baseline for the respective steady-state potentials and to demonstrate that these potentials were present (and present only in the according condition).

In the experimental blocks, participants had to attend to only one of the two modalities at the same time, resulting in the two conditions, here called *attend-to-auditory*, and *attend-to-visual*. To achieve this, participants were cued at the beginning of each block as well as at random times during blocks by a bimodal stimulus signaling a switch in modality. For this, the rectangle changed its color to pink (L * = 60, a* = 80, b* = 13) or turquoise (L* = 60, a* = −28, b* = −13). Which color signaled a switch to which modality was counterbalanced across participants. The task cue was shown for 2 s, with every other color shown for 1 s. In the auditory domain, concurrent with the visual cue, one of two distinct tones could be heard, depending on the to-be-attended-to modality (cues were always presented in both modalities). How often certain auditory and visual stimuli appeared as well as the number of switches to both domains (40 for each domain) was counterbalanced across blocks. The auditory task-switching cue was presented for 2 s, synchronized with the visual task-switching cue.

#### Data recording, processing, and analysis

##### Behavior

We conducted a 2 × 2 × 2 mixed analysis of variance (ANOVA), with *Measurement* (pre/post), *Condition* (visual/auditory), and *Group* (intervention/control) as independent variables on participants’ false alarm rates (defined as number of false alarms divided by the total of all stimuli that could have provoked a false alarm) as dependent variable. We used partial eta squared (eta^2^) as the effect size measure as well as a significance level of alpha (*α*) of 0.05 and planned to follow-up with post-hoc *t* tests in case of a significant interventional effect. We do not report RT and AR analysis because the experiment was not designed to measure classical switch costs (i.e., overt responses were not that frequent and given only to the second of two similar stimuli following a switch cue, i.e., at least 3 s after a switch cue).

##### Electroencephalography recording and preprocessing

EEG data was recorded using an ActiveTwo Biosemi™ electrode system (BioSemi B.V., Amsterdam, Netherlands), at 512 Hz. From 132 electrodes in total, 128 were scalp electrodes, mounted in an equiradial ABC layout. Out of the remaining electrodes, two measured the horizontal and vertical electro-oculogram on the right side and two, located bilaterally on the mastoids, were offline reference electrodes.

EEG data was analyzed using MATLAB (The MathWorks Inc., 2022) and preprocessed with EEGLab (version 2022.1; Delorme and Makeig, 2004) using custom code. Two datasets had to be excluded due to corruption. First, data were resampled to a sampling rate of 256 Hz, after which they were low-pass (1 Hz) and high-pass (40 Hz) filtered. We then separated data according to condition (visual baseline, auditory baseline, attend-to-visual, attend-to-auditory) and rejected faulty channels manually. Five participants were excluded, as more than 15% of their channels had to be rejected in any of the conditions. Next, we created epochs: For analysis of the spectral power of steady-state visual evoked potentials/auditory steady-state responses (SSVEP/ASSR), independently of switching — we selected the first 10 s after each switch and divided the data into 2-s epochs. This was necessary to provide comparability of data in both modalities as the length of each attend-to-modality-X section was pseudorandomized. For time-frequency analysis, which we used to investigate the time course of spectral changes in the data, especially around the time of switches of the to-be-attended-to modality, we selected epochs of 3 s length, with epochs starting 1 s before and ending 2 s after the time-locking stimulus (here, the cue indicating a modality switch). Epochs containing artefacts other than eye blinks were manually rejected by trained personnel. After exclusions, if more than 25% of epochs in any of the four conditions at pretest or posttest had to be excluded, the participant was excluded from further data analysis entirely (five participants). From the data of the analyzed participants, an average of 4.13% of epochs was rejected. An independent component analysis (ICA) was run on the remaining data to identify components reflecting eye blinks and other artefacts, which were manually rejected. The number of excluded components ranged from zero to six, with a Median of two. Only then did we interpolate rejected channels to not cause rank deficiency problems in ICA due to additional dimensions of previously interpolated channels with a near-zero eigenvalue.

##### Spectral analysis

**Figure 4:**
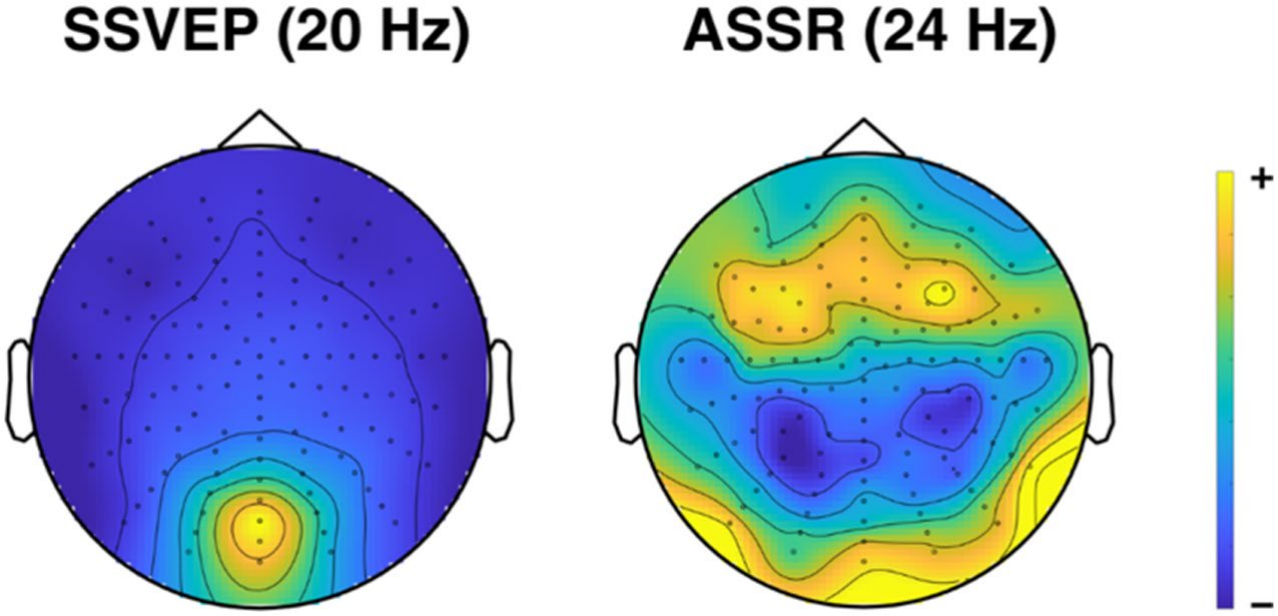
Topographical map of spectral activity for steady-state responses. Topographical distributions of the steady-state visual evoked potential (SSVEP) and the auditory steady-state response (ASSR), averaged across pre- and posttests, groups, and conditions. Yellow areas indicate the highest levels of activity, dark blue areas indicate the lowest levels.

After preprocessing, we conducted a spectral analysis using the FieldTrip toolbox (Oostenveld et al., 2011), to investigate whether there were changes in steady-state responses due to the intervention. For this, raw power was calculated from 1 to 42 Hz, in 1-Hz steps, for each participant at each timepoint, by applying a Fourier transform using multitaper frequency transformation based on discrete prolate spheroidal sequences (Percival and Walden, 1993). To identify steady-state potentials significantly different from noise (Meigen and Bach, 1999), to increase their visibility (Derzsi, 2021), and due to the wide range that participants’ absolute power in distinct frequencies usually takes, we transformed participants’ raw power values into signal-to-noise ratio (SNR). We did this by dividing the raw power values for each frequency band, timepoint, and channel for each participant by the average raw power of the neighboring frequencies (−2 Hz, −1 Hz, +1 Hz, +2 Hz). Further, we subtracted a value of one from the resulting SNR, so that it can be seen as a signal measure relative to the noise level (here, the denominator of the SNR; Figure 4).

For both attend-to-visual and attend-to-auditory conditions, we averaged over those channels where the respective SNR of the steady-state responses was the highest, averaged over pre- and posttests (four occipital channels for SSVEP, 15 frontocentral channels for ASSR).

We then conducted repeated-measures ANOVAs to (1) show that there were steady-state responses present at the stimulation frequencies in the experiment, and (2) test whether the intervention had any effect on the SNR of these steady-state responses (H2a). For (1), we focused on the baseline conditions where only an SSVEP (but not an ASSR) should be present in the visual baseline condition, and only an ASSR (but not an SSVEP) in the auditory baseline condition, and computed three repeated-measures ANOVAs, with *Measurement* (pretest/posttest), *Condition* (attend-auditory/attend visual) and *Group* (intervention/control) as independent variables, and with spectral SNR at posterior-occipital electrodes at 20 Hz and 40 Hz (sensitive to the frequency of the visual stimulus), and at frontocentral electrodes at 24 Hz (sensitive to the auditory stimulus) as dependent variables. We expected to find significant main effects of condition (indicating the sole presence of the appropriate steady-state potential), and no threefold interactions. For (2), we conducted three additional 2 × 2 × 2 mixed ANOVAs for data from the experimental blocks, with *Measurement* (pretest/posttest), *Condition* (attend-auditory/attend visual) and *Group* (intervention/control) as independent variables, and the dependent variables SNR at 20 and 40 Hz at posterior-occipital electrodes, and SNR at 24 Hz at frontocentral electrodes. If the yoga intervention had a significant effect on participants’ SSVEP or ASSR (H2a), we expected to find a threefold interaction.

**Figure 5:**
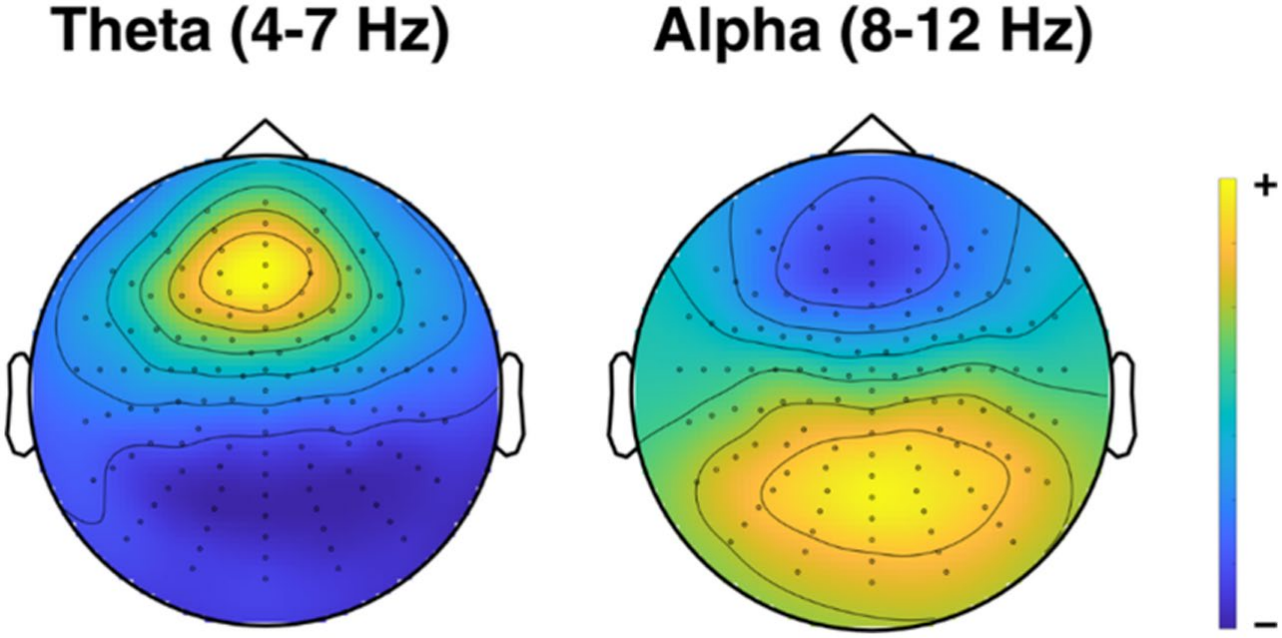
Topographical map of spectral activity for theta and alpha. Topographical distributions of theta- and alpha band activity, averaged across pre- and posttest, groups, and conditions. Yellow areas indicate the highest levels of activity, dark blue areas indicate the lowest levels.

We were, in an exploratory manner, also interested in how posterior alpha as well as frontocentral theta activity has changed due to the intervention. For the selection of the appropriate region, we again averaged over both timepoints and groups, and looked at the spectral distribution of frequencies between 8 and 12 Hz for both attend-auditory and attend-visual conditions for the alpha band, and between 4 to 7 Hz for the theta band. The electrodes with the highest alpha and theta power overlapped across conditions, and we subsequently selected these (alpha: 44 electrodes; theta: 10 electrodes) to measure changes in posterior alpha activity and frontocentral theta activity. Figure 5 shows the topographical distribution of the theta- and alpha-band in our participants, averaged over pre- and posttests.

##### Time-frequency analysis

To test H2b and H3, using the FieldTrip toolbox again, we conducted a time-frequency analysis with a time window of 1 s before, until 2 s after stimulus onset. For this, we used multitaper method convolution with Hanning tapers. The time from 1 s until 0.25 s, before the onset of the cue signalizing a change in to-be-attended-to modality (requiring participants to change their focus of attention from the visual to the auditory modality or vice versa), was used as a baseline, against which we normalized the rest of the data in the epoch by subtracting the mean of the baseline from each data point and then dividing each resulting value by the baseline. By this, we obtained the relative change in power in the current modality compared to the previously attended-to modality. This resulted in a participant × channel × frequency × timepoint matrix, averaged over distinct trials.

We then subtracted pretest from posttest normalized power values for each participant, channel and timepoint, and, using cluster-based permutation tests with 1,000 permutations and an alpha level of 0.05, tested whether there were significant clusters in the data reflecting differences between the intervention and the control group.

For the analysis of SSVEP (20 and 40 Hz) and ASSR frequencies (24 Hz^1^) (H2b), we averaged over the channels where these steady-state responses were present (see spectral analyses above). Regarding theta’s role in aiding task switching (H3), we were interested in (1) whether there were significant clusters showing changed theta activity in the data in response to a switching cue, (2) which distinct frequencies within these frequency bands were particularly affected by these changes, and (3) their exact time course, potentially revealing more about the neural changes that took place due to the intervention aiding task execution or switching. We ran a permutation test for theta (4–7 Hz) activity, including all 128 channels in our analysis, and treating them, just as all timepoints and frequencies, as independent data points. When we found a significant difference, indicative of an effect of the intervention, we followed up calculating contrasts (paired *t* tests) by testing for significant clusters of pre-to-posttest differences in relative changes of power for each group separately, restricted to the timeframe where pre-to-posttest differences between groups (the effect of the intervention) were significant.

## Results

### Experiment 1: Switching attention between colors

Seventy-four participants completed the first experiment during both pretests and posttest. We did not have to exclude any of these participants, so that data of all 74 participants could be analyzed (64 female; *N*_Intervention_ = 34, *M*_Age_ = 24.95 years; *SD*_Age_ 4.48 years; range 18–40 years).

#### Switching costs

Switching costs were present in RTs (in both pre- and posttests), *M* = 8 ms, *t*(73) = 5.61, *p* < 0.001, *d* = 0.65. This is in line with the RT differences found originally by Büsel et al. (2019)—14 ms—, with our standardized effect size being larger (compared to *d* = 0.21) due to the considerably larger number of participants. However, the intervention had no effect on RT switching costs, *M* = 1.49, *t*(73) = 0.40, *p* = 0.346, *d* = 0.09, thus speaking against a role of our hatha yoga intervention on switching. For accuracy rates, we did not find switching costs, *M* = 0.05%, *t*(73) = 0.26, *p* = 0.399, *d* = 0.03. Unsurprisingly, the intervention also had no effect on switching costs in accuracy rates, *M* = −1.10%, *t*(72) = −1.62, *p* = 0.945, *d* = −0.38.

#### Mixing costs

**Figure 6:**
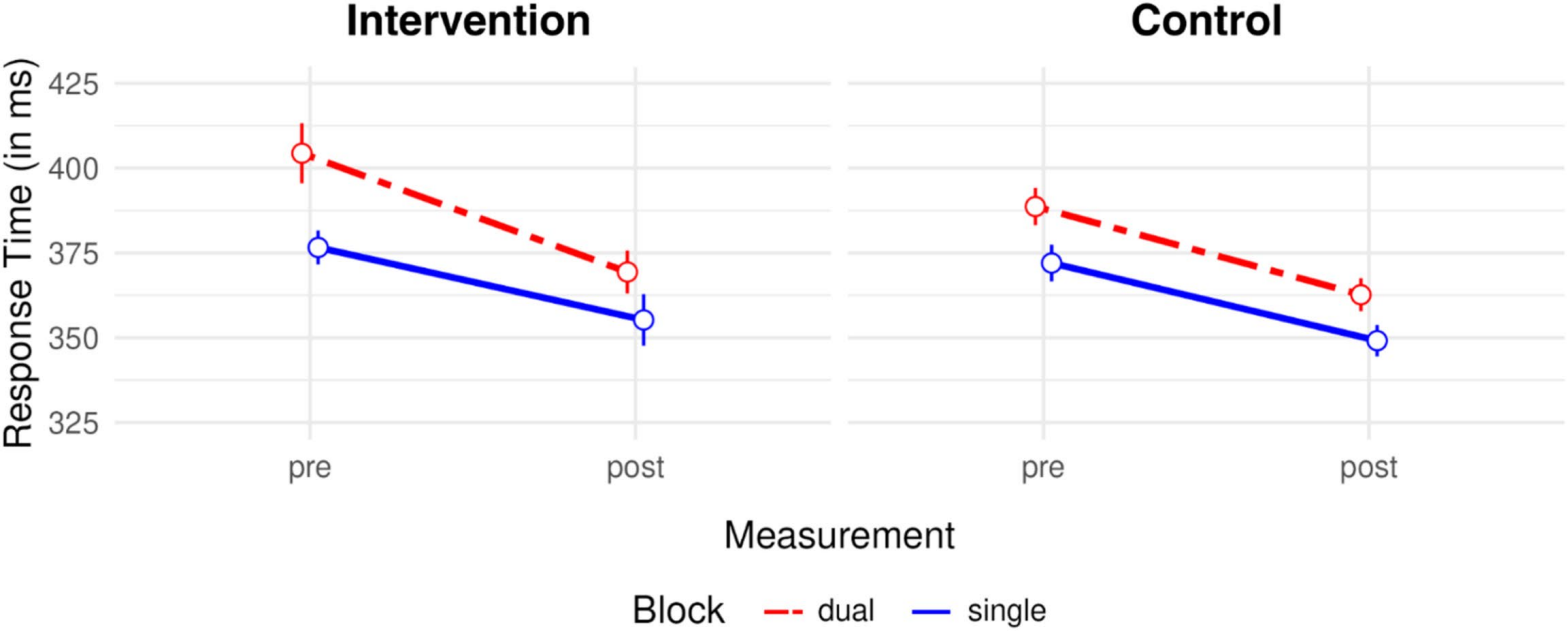
Mixing costs in response times. Dual-color (red dashed line) versus single-color (blue solid line) trial-by-trial target color repetitions as a function of pre- and posttest measurements and groups (intervention vs. control). The difference in response times between dual- and single-color blocks per measurement and group equals the mixing costs. Vertical bars indicate 95% CIs.

As expected, in dual-color blocks, RTs were 18 ms longer than in single-color blocks, *t*(73) = 8.59, *p* < 0.001, *d* = 1.00, reflecting mixing costs. Figure 6 shows mixing costs for RTs. These effects can be seen as large even in spite of the original experiment (*d* = 0.37) as well as the relevant literature. Importantly, mixing costs decreased 10 ms more in the intervention group than in the control group compared to pretest baseline, *t*(72) = 1.75, *p* = 0.042, *d* = 0.41. The direction and magnitude of this reduction suggest that the yoga intervention had a meaningful impact on participants’ ability to efficiently keep multiple feature representations in working memory. These effect sizes could translate to noticeable improvements in daily life, particularly for tasks relying on additional executive resources (although it should be noted that such transfer was not tested here), a point that we will take up again in the discussion.

We also found mixing costs in accuracy rates, *M* = 1.46%, *t*(73) = 4.04, *p* < 0.001, *d* = 0.47. Unlike in RTs, the intervention did not affect mixing costs in ARs, *M* = 0.38%, *t*(72) = 0.25, *p* = 0.403, *d* = 0.06.

### Experiment 2: Task switching between modalities

#### Behavioral results

Sixty-eight participants completed the experiment both during pretests and posttests (with every participant who completed Experiment 1 completing Experiment 2 as well, ensuring a highly overlapping sample). If a participant missed out on too many targets, or if a participant had too many false alarms (as this mostly resulted from not knowing about the correct to-attend-to modality), we excluded them from the experiment. This resulted in nine exclusions altogether, leaving 59 participants (51 female, *N*_Intervention_ = 26, *M*_Age_ = 25.01 years; *SD*_Age_ = 4.57 years; range 19–40 years) for behavioral data analysis.

**Figure 7:**
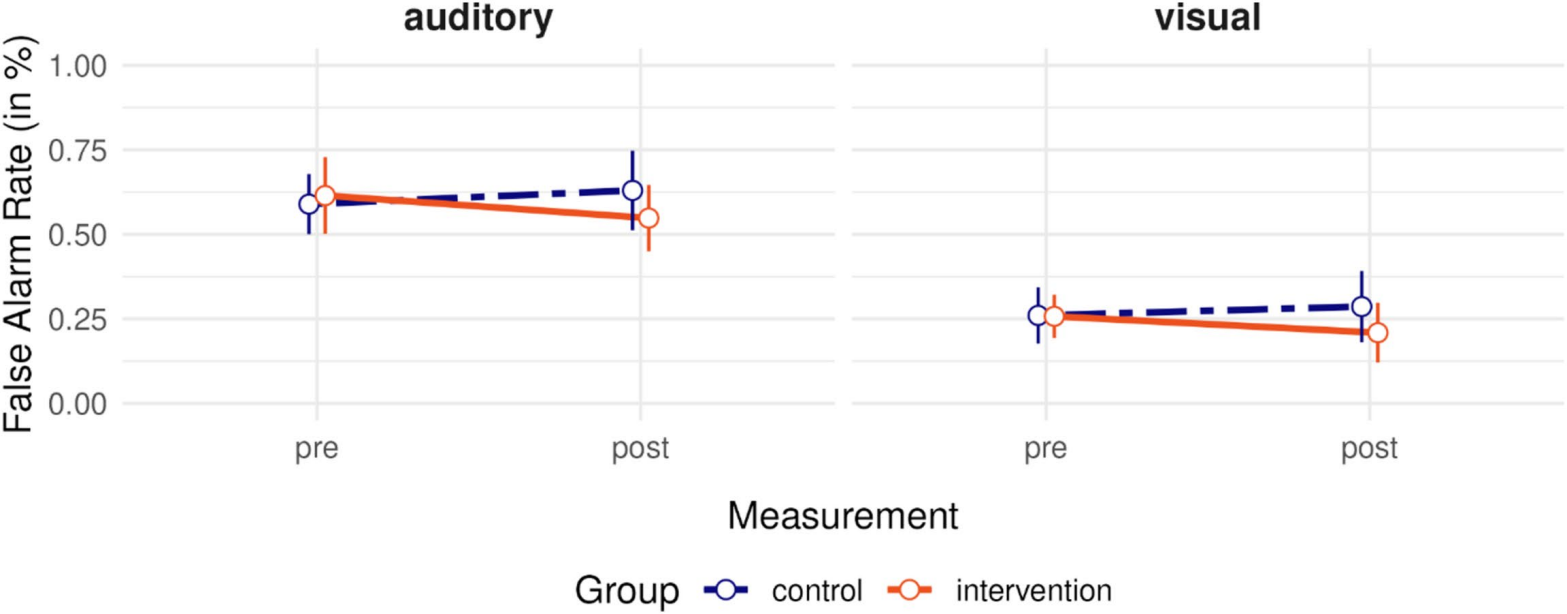
False alarm rates in Experiment 2. False alarm rates shown split by to-be-attended-to modality for both measurement time points (pretest and posttest), as well as for both groups: Orange solid lines for the intervention group, and purple dashed lines for the control group. Vertical bars indicate 95% CIs.

The mixed ANOVA resulted in a significant main effect of Modality, *F*(1, 57) = 63.32, *p* < 0.001, eta_p_^2^ = 0.53, showing significantly more false alarms in case of attend-to-auditory trials, but no significant interaction between group and measurement, *F*(1, 57) = 2.13, *p* = 0.150, eta_p_^2^ = 0.04 (indicating no modality-independent changes in false-alarm rates), as well as no significant interaction between group, measurement, and modality (indicating no modality-specific changes in false alarm rates), *F*(1, 57) = 0.10, *p* = 0.750, eta_p_^2^ < 0.01. All other interactions and main effects were not significant, all *p*s > 0.694. Figure 7 shows false alarm rates for both modalities, groups, and timepoints.

#### Electrophysiology

For the analysis of electrophysiological data, all participants omitted from the behavioral analysis were excluded (for details, see CONSORT chart in Figure 1). Additionally, we had to exclude five participants due to their high numbers of to-be-interpolated channels, as well as five participants due to their high percentages of to-be-deleted epochs. Two datasets could not be used for analysis because they were corrupted, leaving us with the final sample of 47 participants (38 female, *N*_Intervention_ = 21, *M*_Age_ = 24.64 years; *SD*_Age_ = 4.39 years; range 19–40 years) for data analysis.

##### Spectral analysis of steady-state responses

**Figure 8:**
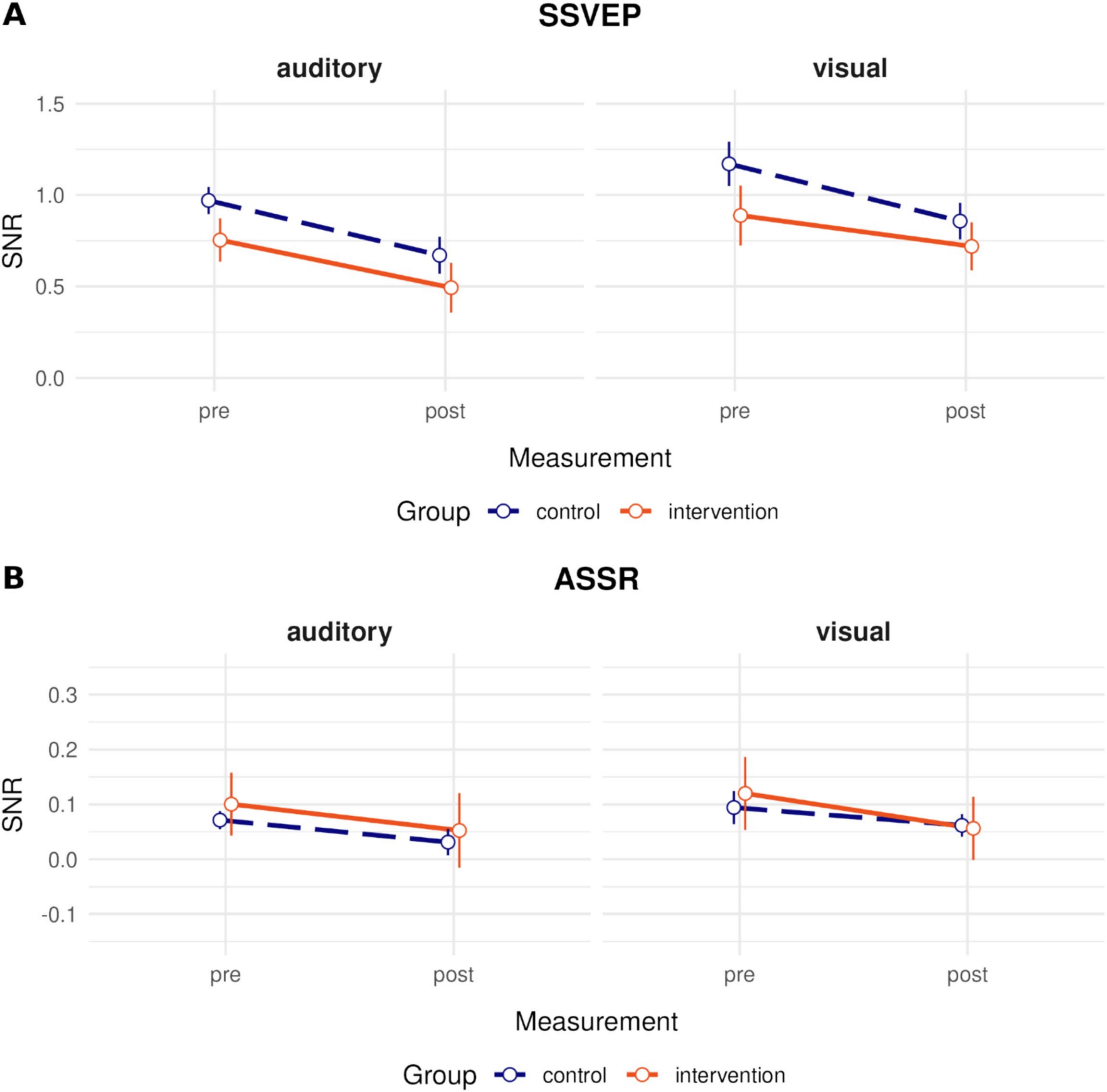
Differences in steady-state responses. Grand average Signal-to-Noise-Ratio (SNR; raw power divided by the mean of the neighboring frequencies minus 1) for **(A)** 20 Hz—the presentation frequency of the visual stimuli, eliciting a Steady-State Visual Evoked Potential (SSVEP); **(B)** 24 Hz—the presentation frequency of the auditory stimuli, leading to an Auditory Steady-State Response (ASSR) for each measurement (pretest/posttest), to-be attended modality (auditory/visual) and group (control = orange solid line/intervention = dark blue dashed line).

We wanted to know if SSVEP and ASSR changed in magnitude between attend-to-auditory and attend-to-visual conditions due to the intervention. After testing for the presence of the steady-state potentials in the first place (as a “sanity check,” all *p*s < 0.001), we tested if the intervention had any effect on them. This was not the case, as reflected in the lacking threefold interaction for SSVEP frequencies, 20 Hz: *F*(1, 45) = 0.47, *p* = 0.273, eta_p_^2^ = 0.03; 40 Hz: *F*(1, 45) = 0.07, *p* = 0.637, eta_p_^2^ < 0.01, and the ASSR frequency, 24 Hz: *F*(1, 45) = 0.99, *p* = 0.325, eta_p_^2^ *= 0*.02. Also, no significant interactions between measurement and group indicative of modality-unspecific steady-state potential changes due to the intervention were found, all *p*s > 0.479. Figure 8 shows the signal-to-noise ratio (SNR) of the first 10 s after each modality switch for all four conditions.

For SSVEP frequencies, there was a significant main effect of measurement, reflecting a decrease of SNRs from pre- to posttest at 20 Hz, *F*(1, 45) = 17.21, *p* < 0.001, eta_p_^2^ = 0.28, and at 40 Hz: *F*(1, 45) = 9.59, *p* = 0.003, eta_p_^2^ = 0.18. However, there was no such decrease of ASSRs at 24 Hz, *F*(1, 45) = 3.03, *p* = 0.089, eta_p_^2^ = 0.01. The main effects of condition were significant at 20 Hz SSVEP, *F*(1, 45) = 34.72, *p* < 0.001, eta_p_^2^ = 0.44, and 40 Hz SSVEP, *F*(1, 45) = 30.88, *p* < 0.001, eta_p_^2^ = 0.41, and for 24 Hz ASSR, *F*(1, 45) = 6.64, *p* = 0.013, eta_p_^2^ = 0.13. These results reflect higher SNRs for 20 Hz and 40 Hz SSVEPs in the attend-to-visual than in the attend-to-auditory condition, and higher ASSRs in the attend-to-auditory than in the attend-to-visual condition. This is plausible because there should be a change in complementary steady-state activity once the stimulus in question is attended to. All other interactions and main effects were not significant at any of the target frequencies (all *p*s > 0.194).

**Figure 9:**
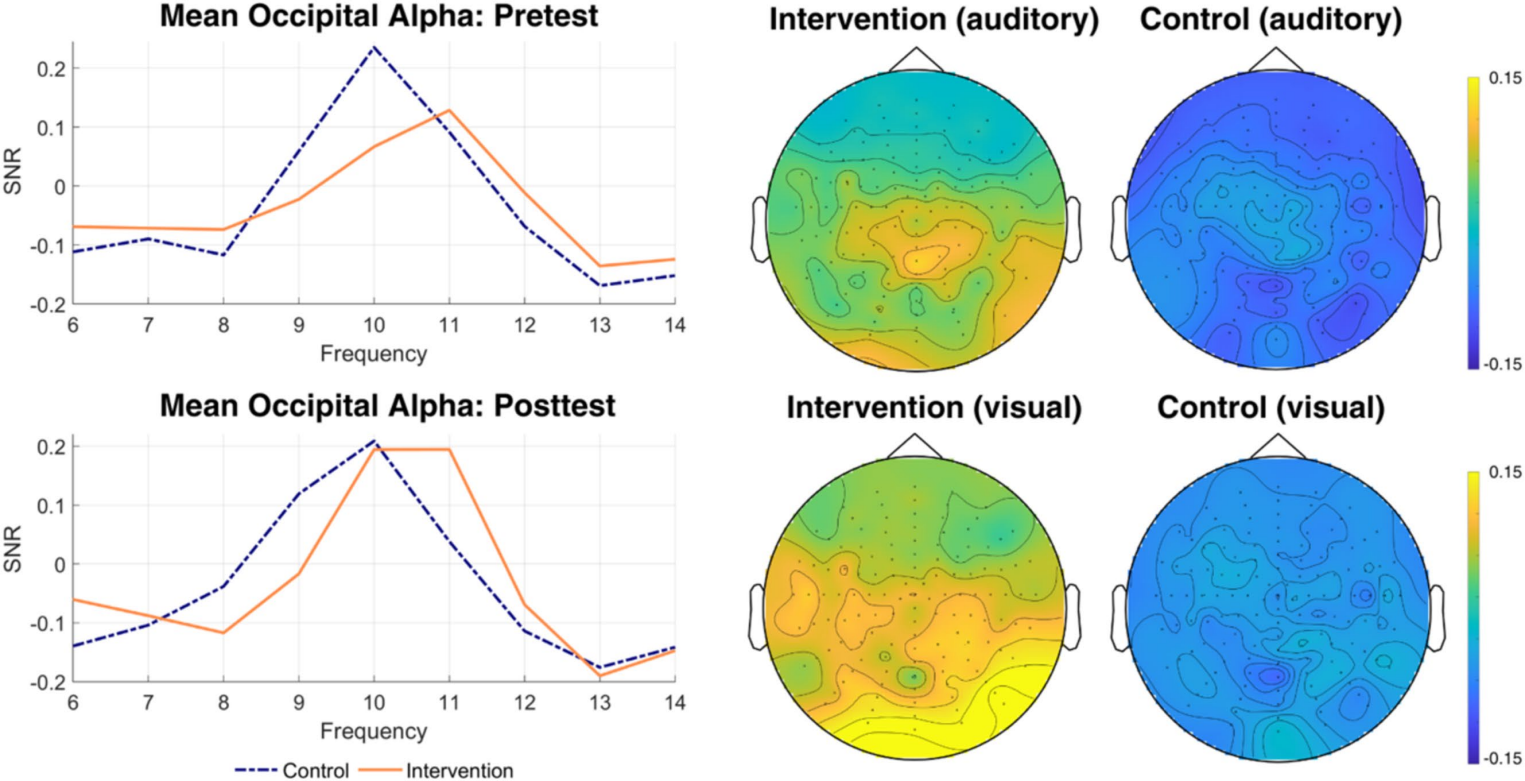
Changes in mean posterior alpha. Spectral distributions for mean posterior alpha (on the left), as well as the topography of changes at 10 and 11 Hz from pretest to posttest by group for the frequencies exhibiting the largest changes. Spectral distributions are shown for both pretest (top) and posttest (bottom), for the intervention (orange solid line), as well as the control group (purple dashed line). The topographical map indicates the locus of changes in the respective alpha rhythm.

**Figure 10:**
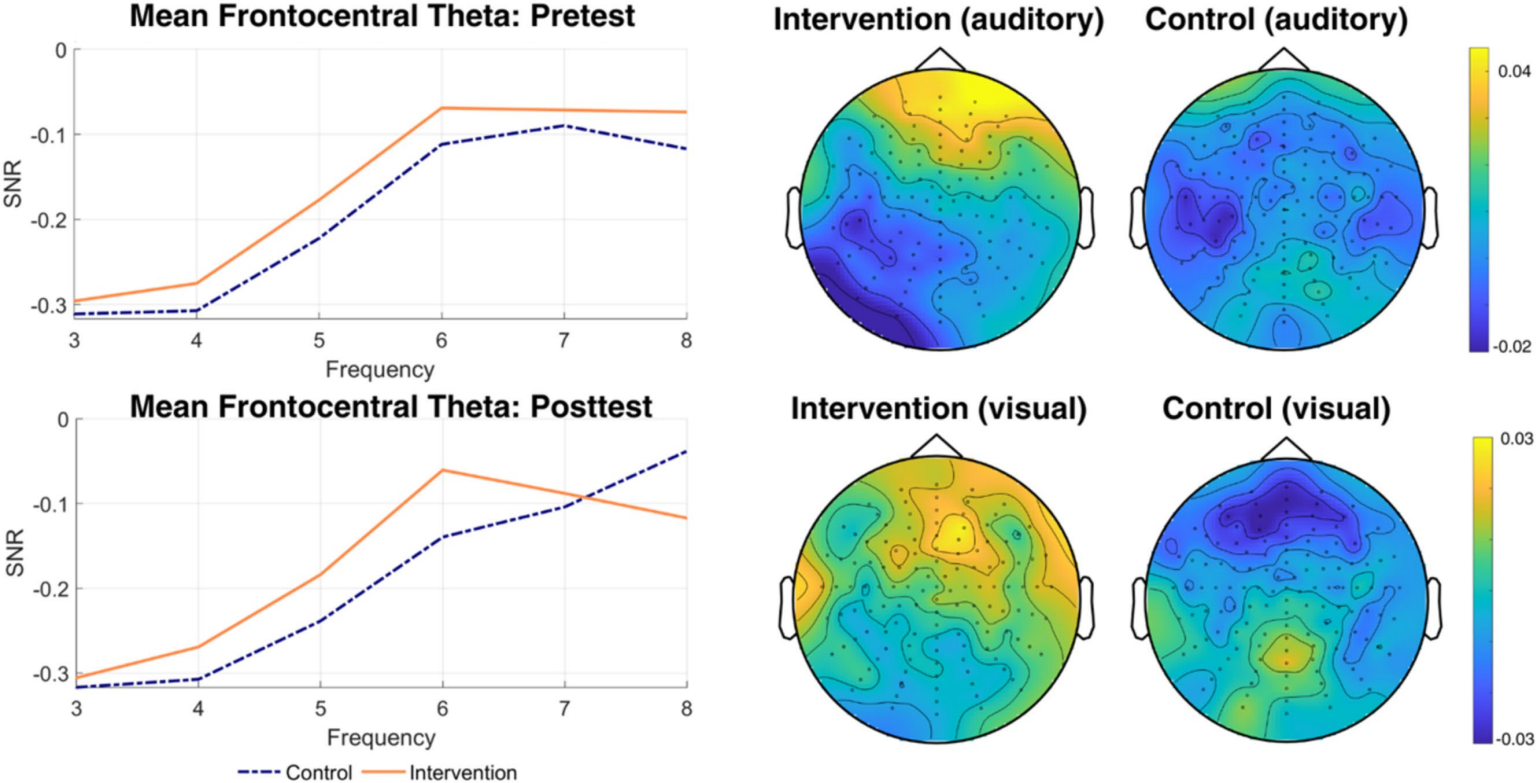
Changes in mean frontocentral theta. Spectral distributions for mean frontocentral theta (left) for pretest (top) and posttest (bottom) for intervention (orange solid line) as well as control (purple dashed line) groups, and the topography of changes from pretest to posttest averaged over the theta band (4–7 Hz), split by group and condition (auditory vs. visual).

Our exploratory analyses further looked at potential changes in frontocentral theta and posterior alpha (shown in Figures 9, 10). For posterior alpha activity, a 2 × 2 × 2 mixed ANOVA, with *Measurement* (pre/post), *Condition* (visual/auditory), and *Group* (intervention/control) as independent variables, on participants’ mean Signal-to-Noise Ratio (SNR) averaged over 8 to 12 Hz as dependent variable showed an interaction between group and measurement, indicating a significant influence of our yoga intervention, *F*(1, 45) = 5.39, *p* = 0.025, eta_p_^2^ = 0.11. Follow-up *t* tests on posttest minus pretest SNR differences showed that the difference stems from alpha increases in the intervention group, *M* = 0.02, *t*(45) = 3.21, *p* = 0.004, *d* = 0.29, while there was no significant change in the control group, *M* = 0.00, *t*(45) = 0.09, *p* = 0.932, *d* = 0.02.

There was also a significant main effect of condition, *F*(1, 45) = 13.70, *p* = 0.001, eta_p_^2^ = 0.23, with lower SNRs in attend-to-visual, *M* = 0.073, *SD* = 0.020, than in attend-to-auditory conditions, *M* = 0.084, *SD* = 0.026. All other interactions were not significant, all *p*s > 0.118.

For the theta-band, we conducted another 2 × 2 × 2 mixed ANOVA with the same variables on participants’ mean SNR averaged over 4 to 7 Hz. Here, we also found an effect of the intervention, reflected in an interaction between group and measurement, *F*(1, 45) = 1.98, *p* = 0.018, eta_p_^2^ = 0.12. A follow-up *t* test between groups on posttest minus pretest SNR differences again confirmed the finding from the ANOVA, *M* = 0.04, *t*(45) = 2.44, *p* = 0.018, *d* = 0.71. This intervention effect was, again, condition-independent, reflected in a nonsignificant threefold interaction, *F*(1, 45) = 0.06, *p* = 0.812, eta_p_^2^ < 0.01. Again, there was a significant main effect of condition, reflecting higher SNR in the attend-to-visual, *M* = −0.078, *SD* = 0.045, than in the attend-to-auditory condition, *M* = −0.103, *SD* = 0.055. All other main effects and interactions were not significant (all *p*s > 0.135).

##### Time-frequency analysis

**Figure 11:**
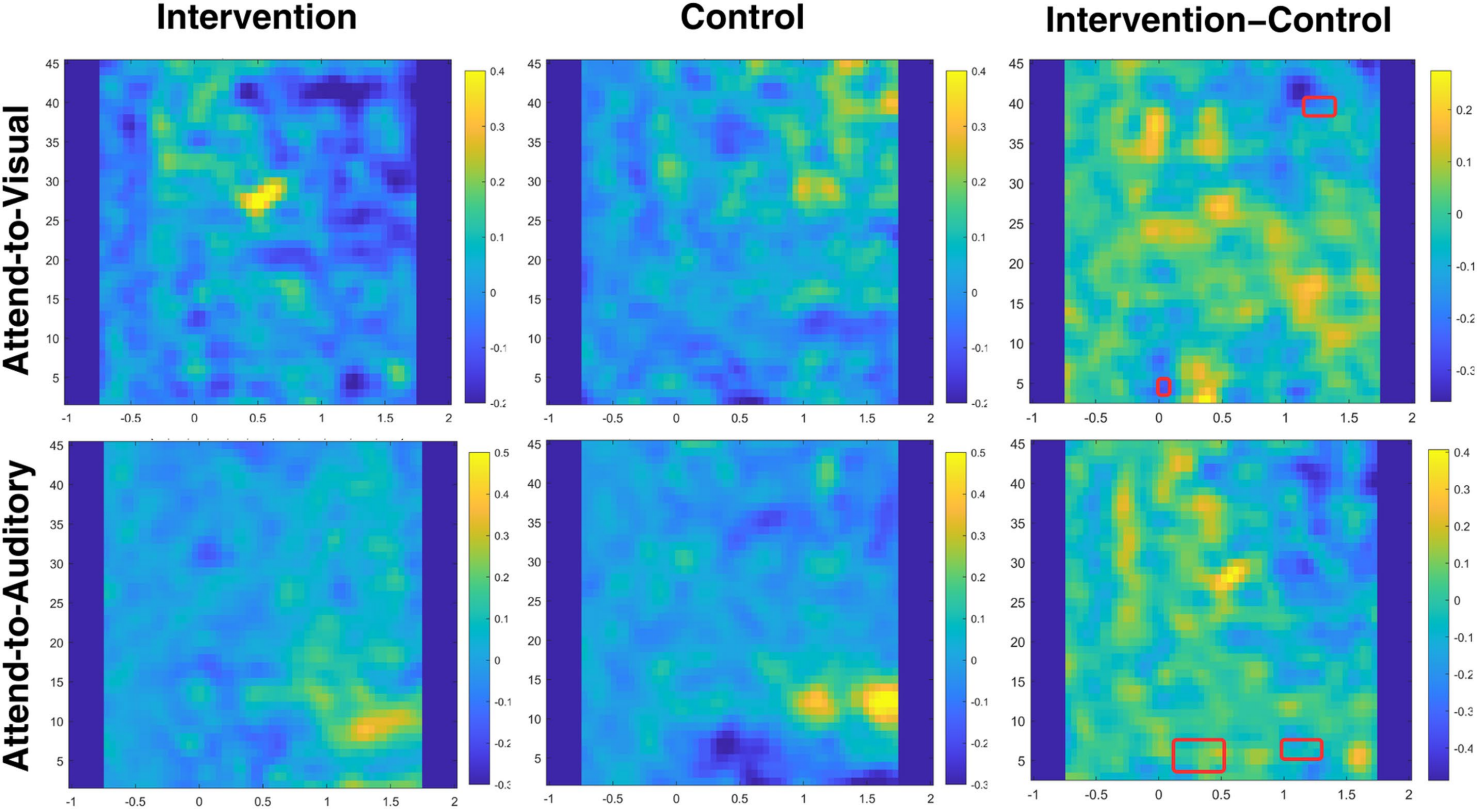
Time-frequency difference plots for all conditions and groups. Time-frequency plots of differences between pre- and post-test values for attend-to-visual and attend-to-auditory conditions for control and intervention groups, as well as differences between groups for both modalities (the rightmost column). The cue indicating a modality switch is the time-locking stimulus. The *x*-axis represents time (from 1 s before cue to 2 s after the cue), and the *y*-axis represents frequency (from 2 to 45 Hz). Brighter colors indicate higher relative change in power at a given frequency and timepoint compared to baseline (the previously attended-to condition). Significant clusters are marked with red filet rectangles.

Figure 11 shows time-frequency plots for both conditions and groups.^2^ We found a significant negative cluster (*p* = 0.017) at 40 Hz between 1,150 and 1,400 ms after switching to the visual modality, reflecting decreased 40 Hz SSVEP (compared to the attend-to-auditory baseline) in the intervention group relative to the control group and pretests. To evaluate the nature of this effect, we conducted two additional cluster-based permutation tests on the pre-to-post-test differences, separately for each group, limited to the timeframe of the significant interaction cluster: While the control group showed a significant increase between 1,250 and 1,400 ms (*p* = 0.010), there were no significant clusters in the intervention group, potentially indicating reduced reactivity to the entraining stimulus. No significant clusters at 20 or 24 Hz were found (all *p*s > 0.103).

**Figure 12:**
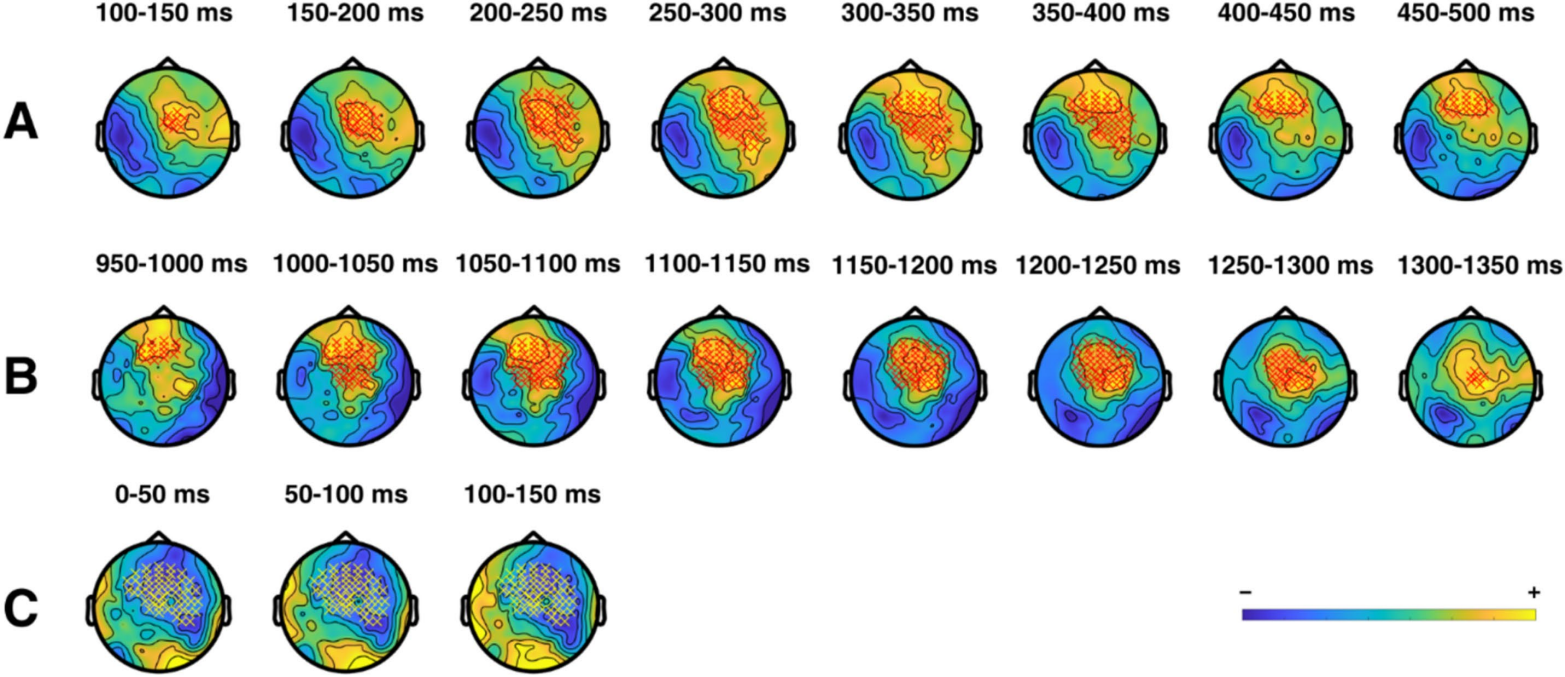
Significant clusters of theta activity. Depicted are significant clusters of electrodes, timepoints, and frequencies, indicating a change in theta power after a switch in modality. Clusters A and B (shown for 7 Hz) show greater theta power in the intervention group relative to the control group after a switch to the attend-to-auditory condition relative to a switch to the attend-to-visual condition. Cluster C (shown for 5 Hz) shows lower theta power in the intervention group compared to the control group after switching to the visual domain relative to switching to the auditory domain.

In our analyses regarding switch-related changes in theta (H3), we found two positive clusters in the data, indicating an increase of a relative change in theta power in the attend-to-auditory trials compared to the respective preceding attend-to-visual trials as baseline, both centered around frontomedial electrodes. Figure 12 shows the time course and topography of all significant interaction clusters. The time course of these changes was from 150 to 500 ms (*p* = 0.035), and from 1,000 to 1,300 ms (*p* = 0.026) after switching from attend-to-visual to attend-to-auditory. Interestingly, these changes were mainly present in the upper theta band (6–7 Hz): In the first cluster (150–500 ms), they started in the upper band before spreading over to the entire theta band for a short time, to be followed by a recurse to 6–7 Hz only. The second cluster consisted entirely of changes in 6–7 Hz. We calculated post-hoc tests for these clusters by performing cluster-based permutation tests for each group individually, comparing pre-to-post-test differences, and restricting for the time range where the interaction was present. For the time range of the first interaction cluster, we only found significantly decreased theta activity in the control group between 150 and 500 ms after switching, *p* < 0.001, indicating decreased theta activity in the control group, but no changes in the intervention group. For the second significant interaction cluster, we found a concurrent positive cluster only in the intervention group (between 1,000 and 1,250 ms), *p* = 0.004, reflecting relative power increase after switching modalities compared to the control group.

In attend-to-visual trials, we found a significant negative cluster (*p* = 0.037), indicating a stronger relative decrease in theta power in the intervention group in the first 100 ms after switching, also centered around frontomedial electrodes (albeit more distributed across the scalp than in case of the positive clusters), but at different frequencies, namely 4 and 5 Hz. Post-hoc-tests showed significant clusters in opposing directions for both groups: A relative theta decrease for the intervention group (0–100 ms, *p* = 0.019), as well as a relative theta increase for the control group (0–50 ms after switching, *p* = 0.027).

## Discussion

In the present study, we set out to test if an eight-week Hatha yoga intervention could benefit the switching of attention in two ways: switching between multiple objects, as well as switching between the auditory and visual modalities. By doing so, we were also able to investigate whether the intervention had distinct effects on these two subtypes of attentional shifting. If yoga improved switching between multiple objects, we expected to see decreased behavioral (switching and mixing) costs in Experiment 1; however, only mixing costs decreased due to the intervention, indicating easier retrieval of task sets from working memory. Therefore, H1 could not be confirmed. Additionally, using a bimodal n-back task with frequency tagging to track the course of attention, our analysis revealed no significant differences in spectral power at the presentation frequencies when stimuli in both modalities were tagged with distinct frequencies (H2a), indicating no changes in the switching function. We also expected changes in power at the respective tagging frequency directly after a modality switch (H2b). This hypothesis could only be partly supported (see the discussion on group differences in 40 Hz activity—the second harmonic of the visual presentation frequency—below).

Finally, we also aimed to investigate if the intervention improved related processes of executive control. In line with our expectations, the intervention led to increased clustered midfrontal theta activity in response to a switch in modalities as a result of the intervention (H3). These results are in line with further exploratory analyses that revealed an increase in posterior alpha that could be related to increased suppression of irrelevant information under conditions of high cognitive load.

### Decreased mixing costs as a potential marker of improved working memory retrieval

In Experiment 1, the intervention led to a significant decrease of mixing, but not switching costs: Participants did counter the increased demands when keeping two target colors rather than one target color in mind better when they had participated in the intervention. Because mixing costs are indicative of the use of distinct task sets (Monsell, 2003) but switching ability between distinct task sets was not improved through the intervention, we think effects on mixing costs were unrelated to task switching, despite being a general aid for switching performance (Kiesel et al., 2010; Mayr and Kliegl, 2000). For example, participants in the intervention group might have found it easier to retrieve different color-specific task sets from working memory, but, as switching depends on the implementation rather than just the retrieval of task sets, improved memory in the form of reduced mixing costs might not always decrease switching costs (cf. Koch et al., 2005). It should also be noted that increased frontoparietal theta connectivity has been associated with reduced mixing costs (McKewen et al., 2020, 2021), suggesting a possible link between improved executive functioning and this behavioral measure across Experiments 1 and 2. However, this link remains hypothetical as these effects were not measured concurrently. To summarize, our results suggest that the intervention did not have a direct effect on switching performance. Instead, it appears to have improved executive control, which is relatively independent of switching.

It has to be pointed out that there were significant differences in performance at pretest, as the intervention group was slower on dual-color trials than the control group, making the comparison of mixing cost differences due to the intervention more complicated. For example, part of the interaction might have reflected a regression to the mean. At the same time, the preexisting group difference could indicate a greater potential for improvement in the intervention group, so that the observed effects could therefore reflect a stronger adaptation due to the intervention.

As for the applicability of our findings, these changes could lead to better performance in everyday activities that require flexible adaptation of executive resources, such as managing multiple work tasks, driving in traffic, or, in the context of yoga, keeping multiple instructions (e.g., for more than one body part) in mind at the same time. Although the 10-ms decrease in mixing costs may look negligible for real-world implications, it should be seen relatively to the size of the original effect: Mixing costs during pretests were 22 ms, so a 10-ms decrease in mixing costs is synonymous with a 45% decrease. Effect sizes of mixing costs in general were large, while the corresponding difference due to the intervention is still considered a medium-sized effect; in cognitive research, even small changes in performance metrics such as response times can have significant implications for daily activities. However, it is important to acknowledge that the extent to which these effects translate to real-world improvements remains uncertain, with the ecological validity of these constructs not yet established. While it is tempting to infer that the improvements observed in the lab could enhance shifting in everyday life, further research is needed to determine whether such transfer actually occurs. Additionally, although the number of situations involving the shifting of attention in daily life might suggest that these effects accumulate over time, it does not necessarily imply that lab-based improvements will have real-world relevance. Future research could aim to establish whether effect sizes seen in experimental tasks can be quantitatively related to real-world outcomes. At this stage, we can only propose that while the data show a potential for real-world improvements, the extent to which this potential translates into meaningful practical effects remains unknown.

Do our results rule out a direct effect of practicing switches during Hatha yoga on switching performance in general? Not necessarily. Benefits from practicing task switching for other executive functions have been observed in the past (Karbach and Kray, 2009), also resulting in improvements in other executive functions. However, it is also possible that other facets of yoga practice account for the effect on mixing costs. For example, yoga requires focusing, for maintaining specific poses, regulating breathing, and monitoring thoughts. Focused attention (characterized by reduced mind-wandering and directed attention towards present experiences, fundamental in mindfulness and yoga) may also profit from yoga practice (Luu and Hall, 2016). In turn, practicing mindfulness within yoga may cultivate a mental state where attention is anchored in the current moment, bolstering executive control functions reflected in reduced mixing costs.

The experimental approach used in our study focused primarily on cognitive mechanisms of executive control, perhaps at the expense of understanding the full spectrum of yoga’s effects. Yoga integrates physical postures with breath control, meditation, and related ethical elements, which may collectively contribute to its cognitive and health benefits in an interrelated manner.

Although the synergistically acting mechanisms in yoga may be too complex to draw upon here, a review by Schmalzl et al. (2015) does attempt to shed a light on the complex mechanisms and effects of yoga’s mechanism. The ancient wisdom of yoga tells us that everything is connected, making the notion plausible that yoga’s components cannot be simply dissected into small parts. Wayne and Kaptchuk (2008) provide an exhaustive analysis of the ecology of possible factors contributing to the beneficial effects of another mind–body practice, tai chi, arguing that it should be seen as a complex practice with synergistically working components. This argument is highly applicable to yoga, with its interconnected mechanisms also suggesting a holistic approach. Therefore, while our focus on cognitive control offers important insights, it may be too narrow to fully capture the complexity of yoga’s effects. A more comprehensive research approach that embraces the holistic nature of yoga—integrating its physical, mental, and ethical dimensions—may be necessary to fully understand how its components synergistically contribute to its cognitive and health benefits.

### Hatha yoga does not impact steady-state potentials, but increases frontocentral theta

Experiment 2 had the objective to look at a facet of the switching function that was different from the one handled in Experiment 1: While Experiment 1 was centered around switching between distinct attentional control sets (here: the colors red and green), Experiment 2 did investigate switching between distinct (auditory and visual) modalities, which can also be seen as two distinct tasks as opposed to Experiment 1. Switching between ACS primarily involves reconfiguring cognitive control settings within a single modality (Dombrowe et al., 2011), while modality switching involves additional processes related to shifting attention between different sensory systems (Murray and Wallace, 2012; Soto-Faraco and Spence, 2002; Spence and Driver, 1997); contrary to the switching of attentional control settings, switching between distinct tasks also involves a switch in response categories (Rushworth et al., 2005). By distinguishing between these two types of switching-related processes, we wanted to know whether the yoga intervention would produce changes in one type of switching but not the other, making us able to further disentangle the presumed effects. However, since our results indicated no significant effect of the intervention on the switching function in either case, doing so was not possible in the first place.

Looking at steady-state potentials, we did not find a change in the spectra of these potentials due to the intervention, neither in the visual (20 Hz SSVEP and its 40 Hz harmonic), nor in the auditory modality (24 Hz ASSR). These results, thus, speak against the validity of H2 and a positive effect of yoga on task switching in healthy participants, too. It may be the case that our baseline selection (with the period immediately before the switch to the attended-to modality potentially confounding modality differences) conflated modality and switch-dependent changes; however, this is also similarly the case for other potential baselines in the current experiment. In addition, statistical tests not showing any significant differences between the respective modality baselines speak against such an interpretation.

However, when focusing on changes in spectral activity over time, following modality switches, 40 Hz activity increased only in the control group after a switch to the visual modality. SSVEP decreases have been observed after an 8-week mindfulness meditation course (Schöne et al., 2018), with the authors attributing this decrease to increased efficiency in the use of attentional resources. As the SSVEP’s amplitude also reflects the amount of resources used for perceiving and processing the stimulus, such a change in sensory and attentional regulation may be advantageous for long-term task performance by saving cognitive resources, as well as through more efficient processing (Andersen et al., 2011). In other words, it is possible that only the participants in the control group felt the need to pay more top-down attention to the visual targets following a task shift and that the less effortful processing in the intervention group was just as appropriate, as was suggested by similar task performance in both groups. Thus, there was no decisive evidence for a change in the switching function, but a hint for increased efficiency in stimulus perception.

A related, and particularly interesting finding in Experiment 2 concerns the significant effects of the intervention on frontocentral theta, particularly when participants switched from attend-to-visual to attend-to-auditory conditions (H3), together with findings of the spectral analyses showing that spectral power of theta increased due to the intervention. Theta oscillations in the frontocentral region have been repeatedly linked to improvements in task switching, as well as to related executive control processes: Midfrontal theta activity in response to stimuli is assumed to reflect adjustment to increasing demands in response to novelty, as well as conflict, through endogenous and exogenous action monitoring processes (e.g., Cavanagh et al., 2012; Cavanagh and Frank, 2014; Enriquez-Geppert et al., 2014). Particularly, during response conflict, a situation where executive control is especially needed, a specific pattern of 6 Hz theta activity over medial frontal cortex is present (Cohen, 2014; Nigbur et al., 2012). Midfrontal theta has also been associated with task rule adjustments, with theta peaks reflecting prediction errors and the need for increased executive control during task switches (Verbeke et al., 2021). In our study, the group differences in theta activity observed after a modality switch may reflect an improved deployment of cognitive resources to resolve conflicts. The increased frontocentral theta SNR further supports this interpretation, indicating enhanced utilization of executive resources.

The distinct patterns and periods of increased theta activity post-switching point to two different cognitive processes at play, with the subsequent increase after 1 s (the second significant cluster) possibly indicating sustained attentional processes (which rely heavily on frontomedial theta oscillations in general) maintaining proactive cognitive control due to the intervention (Clayton et al., 2015; Cooper et al., 2015; Cunillera et al., 2012), while decreases in theta in the control group after a switch point to diminished attentional control due to stimulus-driven attentional processes (Corbetta and Shulman, 2002) not present in the intervention group, possibly due to facilitated executive control (Cavanagh and Frank, 2014).

### Increased alpha: suppression under high cognitive load?

In an exploratory analysis, we were also able to demonstrate a general increase in posterior alpha activity. This outcome can reflect distinct, but possibly interrelated processes. One possible interpretation relies on the upregulation of parasympathetic nervous system activity through the intervention (Eda et al., 2020; Trakroo et al., 2013; Zaccaro et al., 2018), resulting in increased relaxation. This could suggest that participants may have achieved a more relaxed and balanced state during (and possibly also away from) the task. However, increased alpha activity in parietal and occipital regions may also reflect the active inhibition of irrelevant sensory information (Jensen and Mazaheri, 2010), which, in turn, can aid optimal task performance (van Zoest et al., 2021; Zanto and Gazzaley, 2009). In our experiment, participants continuously encountered stimuli from both the attended and the non-attended modality; this is particularly interesting because, if posterior alpha reflects increased inhibition of unwanted stimuli rather than improved processing or concomitant relaxation ability, we would expect it to be higher when suppressing unwanted visual rather than auditory input—which was precisely the case in our study (albeit the modality-specific effect was not tied to the intervention). Therefore, it is plausible that the increase of alpha reflects an enhanced cognitive strategy to actively inhibit and ignore this irrelevant input, ensuring that only pertinent information is processed (Clements et al., 2022, 2023).

Significant changes in the lower theta band (4–5 Hz) within the first 100 ms following a switch to the visual modality may also be a result of modality-specific alpha increases due to the intervention indicating suppression of the visual modality (cf. Clements et al., 2022, 2023; Pomper and Chait, 2017; Pomper et al., 2015), with frontal theta serving as the cognitive control signal (Cavanagh and Frank, 2014; Cooper et al., 2019). As our behavioral performance decrements for attend-to-auditory conditions (see also Pomper et al., 2023) suggest an asymmetrically increased necessity for modality inhibition, switching to the visual modality may have led to the liberation of frontal areas. Thus, the intervention may have made it possible for participants to employ increasingly more resources to maintain executive control, even under difficult conditions (Duncan, 2010).

### Limitations, challenges, and future directions

By using both behavioral and neural indices of participants’ switching abilities, we were able to provide an objective answer to the question of whether Hatha yoga increased participants’ ability to switch their attention more efficiently between tasks and modalities. The likely answer is “no,” with our results speaking instead for improved executive control processes that are independent from switching. However, there were some limitations to the study that need to be acknowledged in order to provide a complete picture.

The physical nature of yoga, involving various postures that require strength, flexibility, and balance, might be partly responsible for the above-mentioned behavioral and electrophysiological changes: Physical activity has been repeatedly linked to improved executive functioning (Hillman et al., 2003, 2006, 2008). Thus, it is possible that physical activity overshadowed some of the direct improvements in executive functioning through yoga, such as improved switching, and led to the currently found effects. We think that at least in yoga practice, physical and cognitive abilities are practiced jointly and are not improved in a mutually independent manner. However, this could only be demonstrated with an active control group with equivalent levels of physical practice but without the same degree of practice related to executive functioning (cf. Deng et al., 2022; Sharma et al., 2014), the lack of which is, thus, a limitation of the present study.

A further limitation of our study is the moderate training duration of 8 weeks; while extending beyond an introductory phase, it may not be sufficient for developing generalized executive control capacities. The eight-week period was chosen based on previous research showing that short-term interventions can lead to measurable changes in task-switching performance (Gothe et al., 2014, 2016; Strobach et al., 2012) and for feasibility and adherence considerations for a general population. However, developing executive functions, especially for complex tasks like task switching, may require longer engagement. Studies on mindfulness meditation (which shares elements with yoga) suggest that significant cognitive improvements may continue over several months of practice (Hölzel et al., 2011). The moderate duration of our intervention thus might have produced initial improvements, but more substantial gains may require a longer intervention.

Another limitation of our study is the high number of dropouts and exclusions. Despite this, our diverse methods enabled us to draw meaningful conclusions. It was especially important for us to apply rigorous cutoff criteria (e.g., excluding participants with insufficient intervention participation or those who did not understand or comply with the task), which reduced statistical power but increased the internal validity of our measures. Nevertheless, despite the high dropout rate, the statistical power for most analyses was adequate due to the robust repeated-measures design, sufficient to detect at least medium effect sizes, as our sample size calculations were based on the weakest links in power (the questionnaires reported in Szaszkó et al., 2023).

Finally, an important factor that limits the generalizability of our findings was our predominantly female (over 80%), as well as relatively young sample, which consisted mostly of students, with less than a quarter working more than 20 h. Also, some level of self-selection bias, despite our admission restrictions, could have had an influence on the results, with people longing for a change in daily habits or health improvement, signing up for the study. We therefore suspected a relatively high average level of cognitive performance that probably limited any potential effects that the intervention may have had in our study in comparison to tests with a more varied sample. At the same time, this rationale implies that the current intervention effects were measured under conservative conditions and, thus, that the intervention must have been relatively powerful to exert any effect at all. Be that as it may, the insights derived from our study offer a foundation upon which further investigations linking yoga with executive function can be built. We hope our findings encourage researchers to delve deeper into understanding these relationships, especially with the intention of improving people’s mental and physical health.

## Conclusion

We could not observe direct effects of an 8-week Hatha yoga intervention on participants’ switching performance, measured by both behavioral switching costs (in Experiment 1) and changes in oscillatory activity (in Experiment 2). However, the intervention increased participants’ frontocentral theta activity, with frontocentral theta also increasing directly after switching modalities; in line with these results, it also decreased the behavioral costs of keeping multiple target features in working memory. A possible explanation consistent with these findings is an improvement in executive control and switching efficiency (ultimately benefiting switching performance, despite not being directly related to it); however, it is important to clarify that this connection remains hypothetical. The behavioral improvements observed in Experiment 1 and the neural changes in Experiment 2 stem from different experiments with different tasks, and thus, a direct causal link between the two cannot be conclusively established within the scope of this study.

## Data Availability

The raw data supporting the conclusions of this article will be made available by the authors, without undue reservation.

## Footnotes

^1^The second harmonic of the ASSR frequency (48 Hz) was not measured due to an overlap with the line noise frequency.

^2^To test that the selection of our baseline did not negatively influence detection of a switch-related effect and reflected modality-specific differences only, we compared the baselines of both modalities statistically for each frequency (band) of interest and found no differences.
