# Supplementary material for: Testing the impact of hatha yoga on task switching: a randomized controlled trial
Source: Front Hum Neurosci. 2024 Nov 5;18:1438017. doi: 10.3389/fnhum.2024.1438017 (PMC11577087; doi:10.3389/fnhum.2024.1438017)
Supplement: Supplementary file 1 [file Data_Sheet_1.docx]

**Appendix A**

To test if participants indeed used different colors for their top-down sets (i.e., supporting our interpretation of switching costs as costs for changes between top-down sets), we tested solely for the presence of a classical top-down contingent capture effect: a selective or at least higher validity effect under matching than nonmatching conditions in dual-color blocks. To this end, we conducted a 2 × 2 × 2 ANOVA, with the factors *Top-Down Match* (matching/nonmatching) and *Validity* (valid/invalid), plus *Measurement* (pre/post) - the latter to check if our assumptions held both before and after the intervention.

## Response Times

Importantly, the interaction between Top-Down Match and Validity was significant, *F*(1, 73) = 130.24, *p* < .001, eta_p_^2^ = .64, indicating a contingent capture effect, with faster RTs for valid than for invalid matching cues, *M* = 23 ms, *t*(73) = 10.10, *p* < .001, *d* = 0.50. On the contrary, nonmatching cues did not generate such an effect, with 7 ms slower RTs in valid than in invalid trials, *M* = −7 ms, *t*(73) = −2.96, *p* < .007, *d* = 0.15. The main effect of Validity, *F*(1, 73) = 18.96 *p* < .001, eta_p_^2^ = .21, and Top-Down Match, *F*(1, 73) = 49.47, *p* < .001, eta_p_^2^ = .40, were also significant, mainly due to the interaction effect above. Faster RTs in posttests compared to pretests led to a significant main effect of Measurement, *F*(1, 73) = 99.39, *p* < .001, eta_p_^2^ = .58. All other interactions were not significant, all ps > .148, indicating that contingent capture effects were present in both pretests and posttests.

## Accuracy Rates

For accuracy rates, the threefold interaction between Measurement, Top-Down Match, and Validity was significant, *F*(1, 72) = 4.54, *p* = .036, eta_p_^2^ = .06, indicating differences in contingent capture effects between pretests and posttests. Table 2 shows cueing effects (hit rate differences between valid and invalid trials) for both cue types and measurements. This also explains the significant interaction between Top-Down Match and Validity, *F*(1, 73) = 48.56, *p* < .001, eta_p_^2^ = .40. Contingent capture effects were only present in pretest trials with matching cues, while in posttests, they were failed to reach significance. Adequately, the interaction, between Measurement and Validity was also significant, *F*(1, 73) = 21.42, *p* < .001, eta_p_^2^ = .23, driven by this threefold interaction.

There were, again, main effects of Top-Down Match, *F*(1, 73) = 25.76, *p* < .001, eta_p_^2^ = .26, as well as Validity, *F*(1, 73) = 6.21, *p* = .015, eta_p_^2^ = .08. The effect of Measurement was significant as well due to a learning effect, *F*(1, 73) = 23.85, *p* < .001, eta_p_^2^ = .25. The interaction between Measurement and Validity was not significant, *F*(1, 73) = 2.99, *p* = .088, eta_p_^2^ = .04.

| Contrast | *M* | *t*(73) | *p* | *d* |
| --- | --- | --- | --- | --- |
| Pretest: Match | 4.45% | 8.00 | < .001* | 0.64 |
| Posttest: Match | 1.13% | 2.03 | .086 | 0.16 |
| Pretest: Nonmatch | −0.43% | −0.77 | .443 | −0.06 |
| Posttest: Nonmatch | −1.75% | −3.14 | .008* | −0.25 |

*Table A1. Paired t Tests of Cueing Effects by Measurement and Top-Down Match.*
*Note.* Paired *t* tests between valid and invalid trials for pretests and posttests, as well as matching and nonmatching cues. Positive values indicate better performance in valid than in invalid trials (cueing effects). Results with a *p* value < .05 are marked with *.
